# Temporal and functional interrelationships between bacterioplankton communities and the development of a toxigenic *Microcystis* bloom in a lowland European reservoir

**DOI:** 10.1038/s41598-022-23671-2

**Published:** 2022-11-11

**Authors:** Joanna Mankiewicz-Boczek, Arnoldo Font-Nájera

**Affiliations:** European Regional Centre for Ecohydrology of the Polish Academy of Sciences, Tylna 3, 90-364 Łódź, Poland

**Keywords:** Freshwater ecology, Microbial ecology, Natural hazards, Applied microbiology, Microbiome, Environmental biotechnology

## Abstract

The cyanobacteria-associated microbiome is constantly reshaped by bloom development. However, the synergistic-antagonistic nature of the relationships between *Microcystis* and its microbiome still remains unclear. Therefore, temporal changes of bacterioplankton communities and their functional potential through different developing stages of a *Microcystis* toxigenic bloom were investigated, considering bacterioplankton assemblages as particle-attached (PAB) and free-living (FLB) bacteria. 16S rRNA sequencing revealed that PAB were represented by Proteobacteria and Cyanobacteria, while FLB by Proteobacteria and Actinobacteria. Network and ordination analyses indicated that PAB inter-relationships were more complex—numerous connections between taxa with stronger correlations, than FLB—rather influenced by physico-chemical parameters. PAB in pre-summer was diverse with Proteobacteria containing potential taxa involved in nitrogen-transforming processes. In mid-summer, PAB presented a mix-bloom dominated by *Snowella*, *Aphanizomenon*, and *Microcystis*, which were succeeded by toxigenic *Microcystis* in post-summer. Both periods were associated to potential taxa with parasitic/predatory lifestyles against cyanobacteria. In post-summer, Sutterellaceae were recognized as poor water quality indicators, and their strong association with *Microcystis* could have represented an increased threat for that period. *Microcystis* was a major factor significantly reducing PAB diversity and evenness, suggesting that it negatively influenced bacterioplankton assemblages, probably also altering the overall community functional potential.

## Introduction

Cyanobacterial harmful algal blooms (CyanoHABs) are nowadays more frequently observed, with longer and more intense events being reported worldwide^1,2^. Anthropogenic sources, such as agriculture, urban, and industrial activities, are the most important pressures that add a significant load of nitrogen (N) and phosphorus (P) to the freshwater ecosystems and thus facilitating the appearance of CyanoHABs. Furthermore, global climate change is significantly accelerating the process of anthropogenic eutrophication due to the periodical increase of temperature registered every year^3–5^. In bloom-infested waters, light penetration is often reduced and oxygen is depleted towards the benthos (hypoxia), causing significant damage to biodiversity^6,7^. Many cyanobacterial strains also produce toxins that can severely deteriorate ecosystems and human health^8,9^. Microcystin is probably the most important described freshwater hepatotoxin in the literature (63% of global records), followed by cylindrospermopsin (10%), and the neurotoxins anatoxin (9%) and saxitoxin (8%), among others^10^. *Microcystis* spp. are one of the most common cyanobacteria dominating blooms worldwide. They have been reported in at least 108 countries, from which at least 79 were associated to the production of microcystins, a dangerous hepatotoxin that targets liver cells^2^. Microcystins are bioaccumulated by organisms, and therefore, they can harm biodiversity, negatively affect fisheries, and reduce the use of water for drinking purposes and irrigation in the agricultural sector^11^. Additionally, intensive blooms with noxious odours could drastically diminish the scenic beauty of freshwater ecosystems affecting navigation, recreation, tourism, and other economic activities^5,12^. Therefore, the demonstrated activity of CyanoHABs makes it necessary to increase our knowledge on the environmental impact in which the blooms occur, in order to better understand their potential threat, and search for possibilities to mitigate their development.

An important element directly or indirectly affecting cyanobacteria are their interactions with other planktonic microorganisms, including bacteria associated with them (particle-attached bacteria—PAB) and others that coexist at the same time as free-living bacteria (FLB). Attached consortia of microorganisms to the mucilaginous cyanobacterial sheath, referred as the phycosphere, play an important role in the development of CyanoHABs, since the cyanobacterial mucilage provides the optimum environment where different ecological inter-relationships occur^13,14^. It has been hypothesized that the exchange of essential compounds, such as vitamins, CO_2_, N, P, and trace elements by bacteria and extracellular polysaccharides released by photosynthetic cyanobacteria, occurs in the form of symbiotic relationships that promote synergia between both communities^15,16^. Other opportunistic bacteria attach to the mucilage to shelter from pelagic grazers or to feed on cyanobacterial exudates without any apparent benefit for cyanobacteria^17^. Associated bacteria have also been identified to utilize microcystin and its congeners as carbon sources, referred to as the microcystin-degrading bacteria, especially when cyanotoxins are released to the water during bloom decay in late summer^18,19^. Predatory bacteria can also affect bloom development with direct cell contact promoting different endo- and epibiotic lysing mechanisms^20^. Furthermore, certain attached bacteria can also produce exudates that have algicidal properties resulting in cyanobacterial cell lysis^21^. Thus, research focused in the description of PAB communities with comparison to FLB may help to elucidate why cyanobacteria are often dominating phytoplankton in freshwater ecosystems exposed to accelerated anthropogenic eutrophication and, by extension, which PAB can support this effect.

Recent studies have included analyses that allow a closer examination of bacterioplankton community assemblages using high-throughput new generation sequencing technologies (NGS). However, most of the research has not significantly differentiated between PAB and FLB, which is a key aspect that has been useful to identify taxa that are directly linked to bloom occurrence dynamics. In the last decade, studies including discrimination of PAB and FLB, during the development of blooms dominated by *Microcystis* spp., were reported in few publications analysing lakes and reservoirs located in China (Asia)^14,22–24^, with fewer studies from other countries, including France (Europe)^25,26^, and USA (America)^19^. In the specific case of Central Europe, PAB and FLB were not differentiated, and *Microcystis* was not reported as the most dominant cyanobacteria in monitored water bodies^27–30^. In general, the overall research suggests that functional richness and diversity of PAB and FLB communities has been recognized, however their ecological interactions, niche occupancy, and co-occurrence patterns during different stage bloom formation events (temporal patterns) are still poorly unknown^22,23^.

Therefore, to address the above mentioned gap, the present study aimed to describe bacterioplankton community dynamics and their functional potential with the use of 16S rRNA high-throughput sequencing, including the influence of selected water parameters, in the Sulejów Reservoir, located in Poland (Central Europe). The reservoir is also known to regularly experience toxigenic blooms of *Microcystis* in summer season. Particular interest was placed on bacterial taxa that could promote the growth of cyanobacteria, but also taxa that could help mitigate their negative effects, such as parasitic, predatory, cyanotoxin degrading, and algicidal bacteria. Bacterial communities were distinguished from PAB and FLB assemblages using a filtering separation technique, with the objective to: (1) describe changes in composition and diversity between the fractions representing different bacterial communities, including cyanobacteria and microcystins toxicity, during three consecutive times in the summer season; (2) investigate spatial and temporal variations between the co-occurrence of these communities and the local environmental factors (oxygen concentration, pH, nutrient availability, among others); (3) and to identify bacterial taxa that have potential functional importance for CyanoHABs.

## Results

### Surface water environmental characterization

Physico-chemical parameters and nutrient concentrations are described in Table 1. Considering the pre-summer period (PRE) as the base line, the highest temperature was observed in mid-summer (MID, 23.4 °C) and the lowest in post-summer period (POST, 16.6 °C). A similar trend was observed for the pH with the highest alkaline value for MID (9.3) and the lowest for POST (7.2). In contrast, conductivity and oxygen concentration were the highest in PRE (305 µS cm^−1^ and 12.3 mg L^−1^, respectively) and the lowest for the POST (273 µS cm^−1^ and 5.6 mg L^−1^, respectively). Furthermore, the concentration of nitrogen ions as NH_4_^+^ and NO_3_^−^ were the lowest in PRE (2.2 and 598 µg L^−1^, respectively), and highest in the POST (14.9 and 683 µg L^−1^, respectively). For the case of PO_4_^3-^, the concentration was the highest in PRE (75 µg L^−1^) when compared to the subsequent MID and POST (23 and 29 µg L^−1^, respectively).

**Table 1:** Environmental parameters of surface water.

Parameter	PRE	MID	POST
Temperature (°C)	16.8	23.4	16.6
pH	8.1	9.3	7.2
Conductivity (µS cm^−1^)	305	291	273
Oxygen (mg L^−1^)	12.3	11.5	5.6
NH_4_ (µg L^−1^)	2.2	1.0	14.9
NO_3_ (µg L^−1^)	598	599	683
PO_4_ (µg L^−1^)	75.1	23.6	29.3

*PRE* pre-summer period, *MID* mid-summer period, *POST* post-summer period.

### Bacterioplankton community composition

A total of 2,835,952 good-quality reads (83.0%) were obtained from an original database containing 3,415,730 raw reads after 16S rRNA sequencing. Furthermore, 2,315,086 reads (67.8%) were classified with 99% similarity to bacteria (Supplementary material Table S1). A total of 2976 OTUs (PAB: 1485 and FLB: 1491) were assigned to 36 phyla, 84 classes, 211 orders, 351 families, and 698 genera. In general, the predominant phyla in PAB were represented by Proteobacteria (41.5%), Cyanobacteria (32.6%), and Bacteroidota (11.0%), and in FLB by Proteobacteria (37.8%) and Actinobacteria (36.6%) (Supplementary material Table S2).

**Figure 1:**
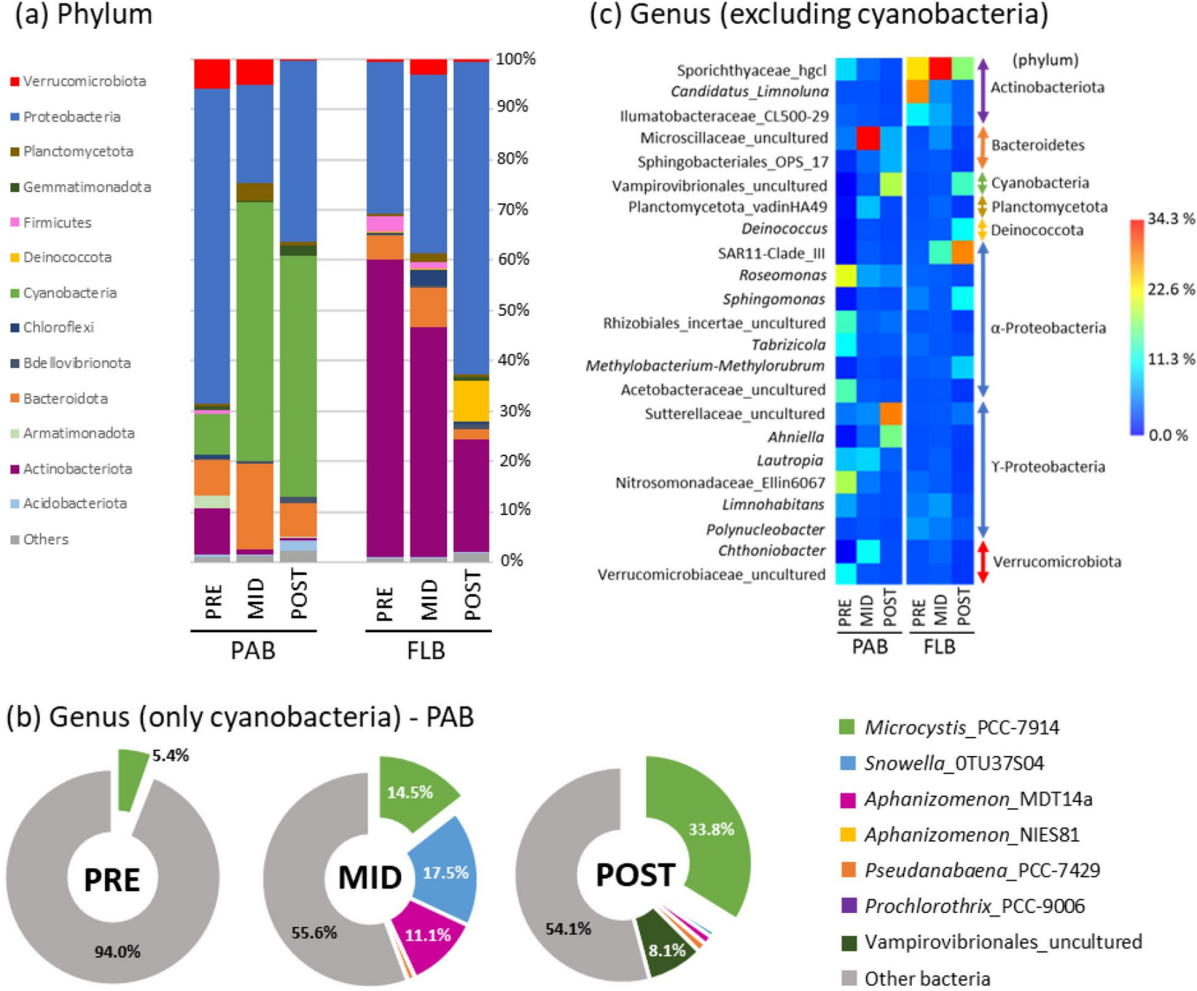
Relative abundance (%) of PAB and FLB communities using 16S rRNA sequencing during the development of a *Microcystis* toxigenic bloom in summer. Overall relative abundances for each taxa are average estimates of relative abundances obtained across samples per period of collection (5–7 replicates, see Supplementary Table S1). Abundances were described to the level of Phylum (**a**), and in the case of genera, cyanobacteria were retrieved from PAB communities and analysed separately (**b** and **c**, respectively). Unclassified sequences and other taxa with lower relative abundance (< 3%) were excluded in (**c**). *PAB* particle-attached bacteria, *FLB* free-living bacteria, *PRE* pre-summer period, *MID* mid-summer period, *POST* post-summer period.

Classified reads are visualized as the total relative abundance of the gene 16S rRNA representing different bacterioplankton communities for the summer season in Fig. 1 and Table S3. For PAB community, Proteobacteria was the most abundant phylum in PRE (62.5%), while Cyanobacteria increased considerably in the subsequent MID (51.4%) and POST (47.9%). Other phyla included Bacteroidota and Planctomycetota with their highest increase in MID (17.1% and 3.5%, respectively) (Fig. 1a). In the case of genus, cyanobacteria were removed from the PAB consortia and analysed separately, in order to investigate the relative weight importance (%) of attached-bacteria without cyanobacteria. The results indicated that cyanobacteria were the most abundant in MID with three genera belonging to *Snowella*_OTU37S04 (17.5%), *Microcystis*_PCC7914 (14.5%), and *Aphanizomenon*_MDT14a (11.1%), followed by highest increases of *Microcystis*_PCC7914 and an uncultured strain of Vampirovibrionales during the POST (33.8% and 8.1%, respectively) (Fig. 1b). The separate analysis of attached-bacteria showed that the highest abundances in PRE were represented by Proteobacteria belonging to *Roseomonas* (7.1%), *Tabrizicola* (3.7%), and uncultured strains of Nitrosomonadaceae_Ellin6067 (6.2%), Rhizobiales_Incertae_Sedis (4.6%), and Acetobacteraceae (4.8%). Most abundant genera in MID were represented by *Chthoniobacter* (8.2%) and an uncultured strain of Microscillaceae (31.0%), while the POST was represented by the highest abundances of γ-Proteobacteria belonging *Anhiella* (11.2%) and an uncultured strain of Sutterellaceae (21.1%) (Fig. 1c).

For the FLB community, the phylum Actinobacteriota presented the highest abundance in PRE (56.5%) with a considerably decrease towards the POST (19.9%). In contrast, the Proteobacteria presented the lowest abundance in PRE (29.0%) with a considerable increase towards the POST (55.0%) (Fig. 1a). In the case of genus, the highest abundances of Actinobacteria in PRE were represented by *Candidatus*_*Limnoluna* (24.8%) and Ilumatobacteraceae_CL500-29 (7.6%), however, the Sporichthyaceae_hgcl was more abundant towards MID (34.0%). The Proteobacteria were more abundant in the POST with OTUs belonging to SAR11_CladeIII (20.6%), *Sphingomonas* (7.5%), and *Methylobacterium*-*Methylorubrum* (5.5%) (Fig. 1c).

### Diversity of bacterioplankton communities

**Figure 2:**
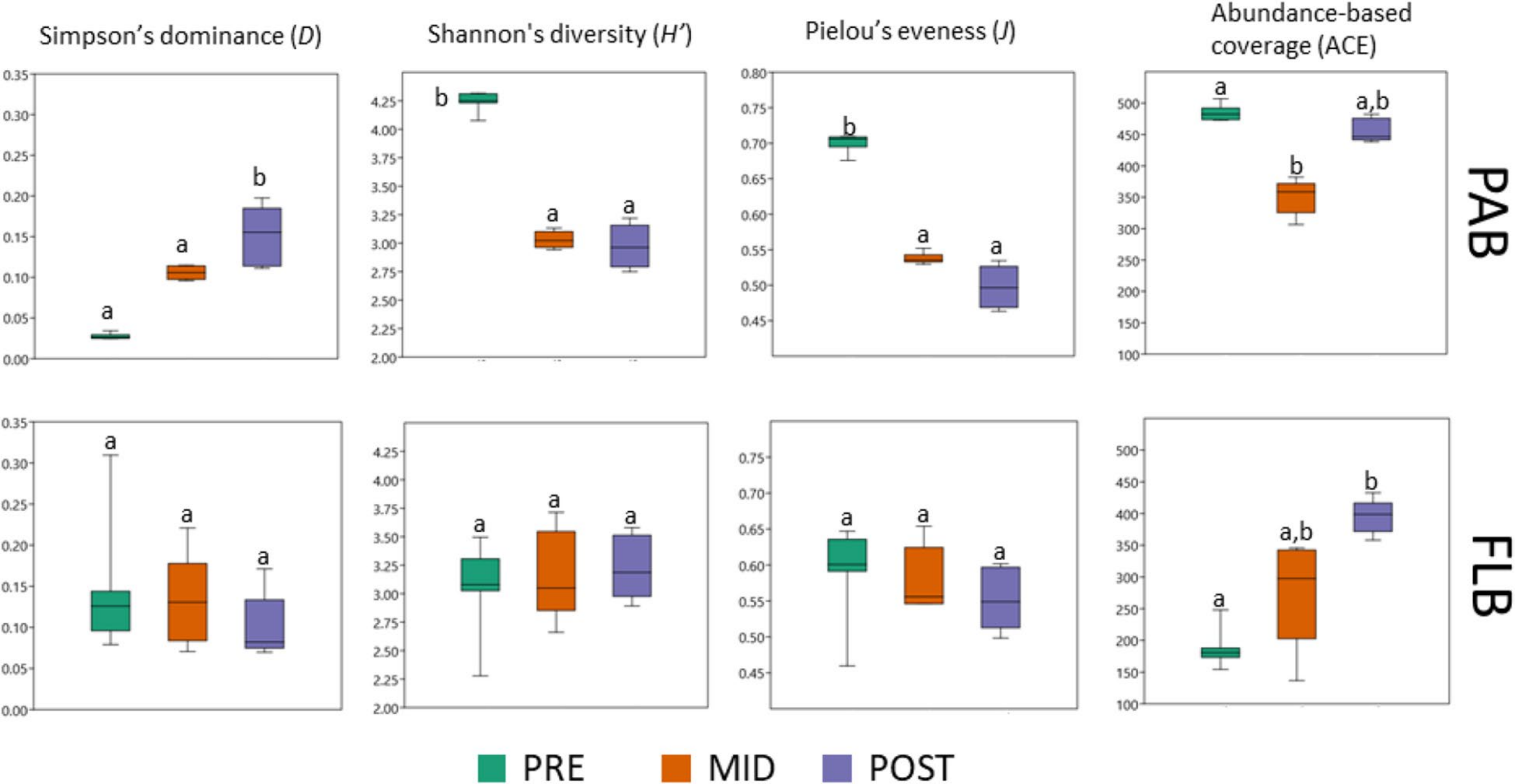
Box-plots representing α-diversity indices for the different bacterioplankton community assemblages. *PAB* particle-attached bacteria, *FLB* free-living bacteria, *PRE* pre-summer period, *MID* mid-summer period, *POST* post-summer period. Significant differences (*p* < 0.01) according to Kruskal Wallis and Dunn’s post-hoc analysis tests were annotated with lower case letters after Bonferroni correction (**a**, **b**).

Diversity indices were summarized in Fig. 2. Rarefaction curve analysis showed that all sample replicates reached a plateau, indicating that the sequencing depth was adequate to capture the overall bacterial diversity (Fig. S1). The PAB showed the lowest value of Simpson’s dominance (*D*) for PRE (0.027 ± 0.003) with a considerable increase towards the POST (0.152 ± 0.030) (Fig. 2). The opposite was observed for Shannon diversity (*H′*), with the highest value observed for PRE (4.246 ± 0.071) followed by a considerable decrease towards the POST (2.991 ± 0.157) (Fig. 2). The above results indicated that PAB community in POST was significantly higher in dominance with respect to PRE (*H* = 16.48, 2 d.f, *p* = 0.000264; see Fig. 2 and Table S4), and PRE was significantly higher in Shannon diversity when compared to MID and POST (*H* = 13.45, 2 d.f, *p* = 0.00220; see Fig. 2 and Table S4). The Pielou’s index (*J*) reflected similar results with Shannon diversity, and the abundance-based coverage (ACE) indicated that there was a similar richness between the PRE and POST (Fig. 2 and Table S4). In the case of FLB community, no significant differences in diversity indices were registered for most sampling periods (*p* > 0.01, see Fig. 2 and Table S4), with the exception of ACE where a significant increase in richness was observed towards the POST when compared to the PRE (*H* = 11.48, 2 d.f, *p* = 0.000216; see Fig. 2 and Table S4).

### Dynamics of toxigenic *Microcystis* bloom formation and microcystins toxicity

**Figure 3:**
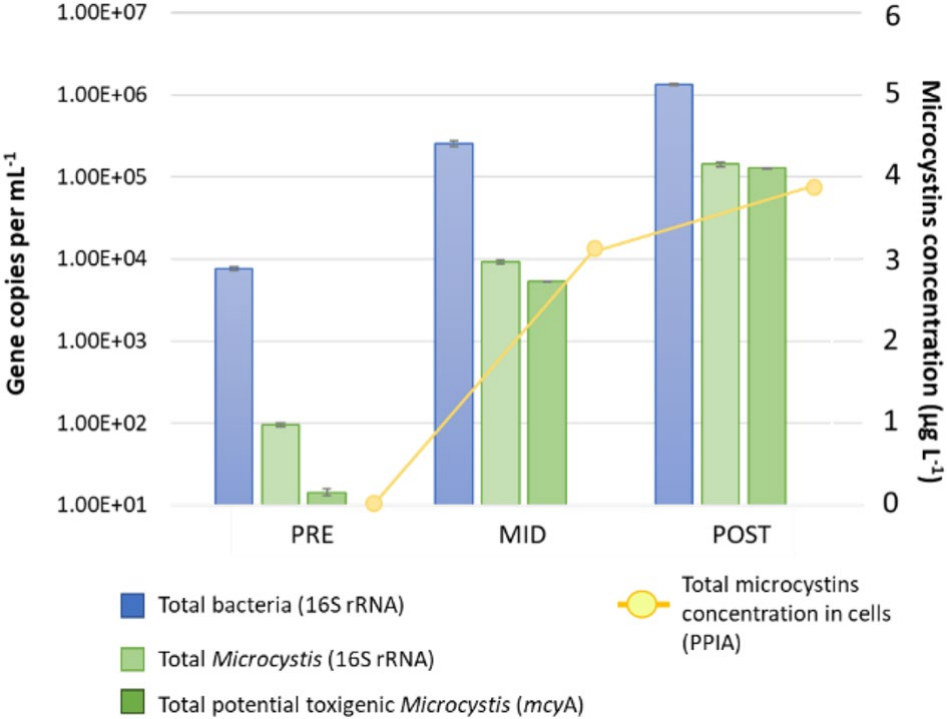
Dynamics of gene abundance and microcytins toxicity (µg L^−1^) in particle-attached bacteria sample fractions (PAB). *PRE* pre-summer period, *MID* mid-summer period, *POST* post-summer period.

The gene copy numbers for PAB assemblages are visualized in Fig. 3 and described in supplementary Table S5. The gene 16S rRNA representing total bacteria showed the lowest copy numbers in PRE (7.60 × 10^3^ ± 385.0 copies mL^−1^) with increasing abundance towards the POST (1.34 × 10^6^ ± 4.42 × 10^4^ copies mL^−1^) (Fig. 3 and Table S5). Similarly, the dynamics of gene copy numbers for the 16S rRNA representing total *Microcystis* spp., and the *mcy*A representing toxigenic *Microcystis* spp., showed their lowest abundances in PRE (95.1 ± 4.5 and 14.3 ± 1.4 copies mL^−1^, respectively) with a considerable increase towards the POST (1.42 × 10^5^ ± 1.09 × 10^4^ and 1.27 × 10^4^ ± 2.76 × 10^3^ copies mL^−1^, respectively) (Fig. 3 and Table S5). The abundance of total bacteria was 80 times higher than that of the total *Microcystis* spp. during PRE, which was considerably reduced to only 9 times difference in the POST (Table S5). Similarly, the abundance of total *Microcystis* spp. was almost 7 times higher than that of the amount of toxigenic strains of *Microcystis* spp. during PRE, which was reduced approximately to a 1:1 ratio for the POST (Table S5). Furthermore, MCs toxicity expressed as microcystins content in the cells was also included in Fig. 3. MCs were detected in MID (3.21 µg L^−1^) and in POST (3.87 µg L^−1^). The microcystins concentration (toxicity) correlated to the *mcy*A gene copy numbers (*r*^*2*^ = 0.98), corroborating that the POST bloom was dominated by toxigenic *Microcystis* populations (Fig. 3).

### Relationships between bacterial communities and environmental parameters

**Figure 4:**
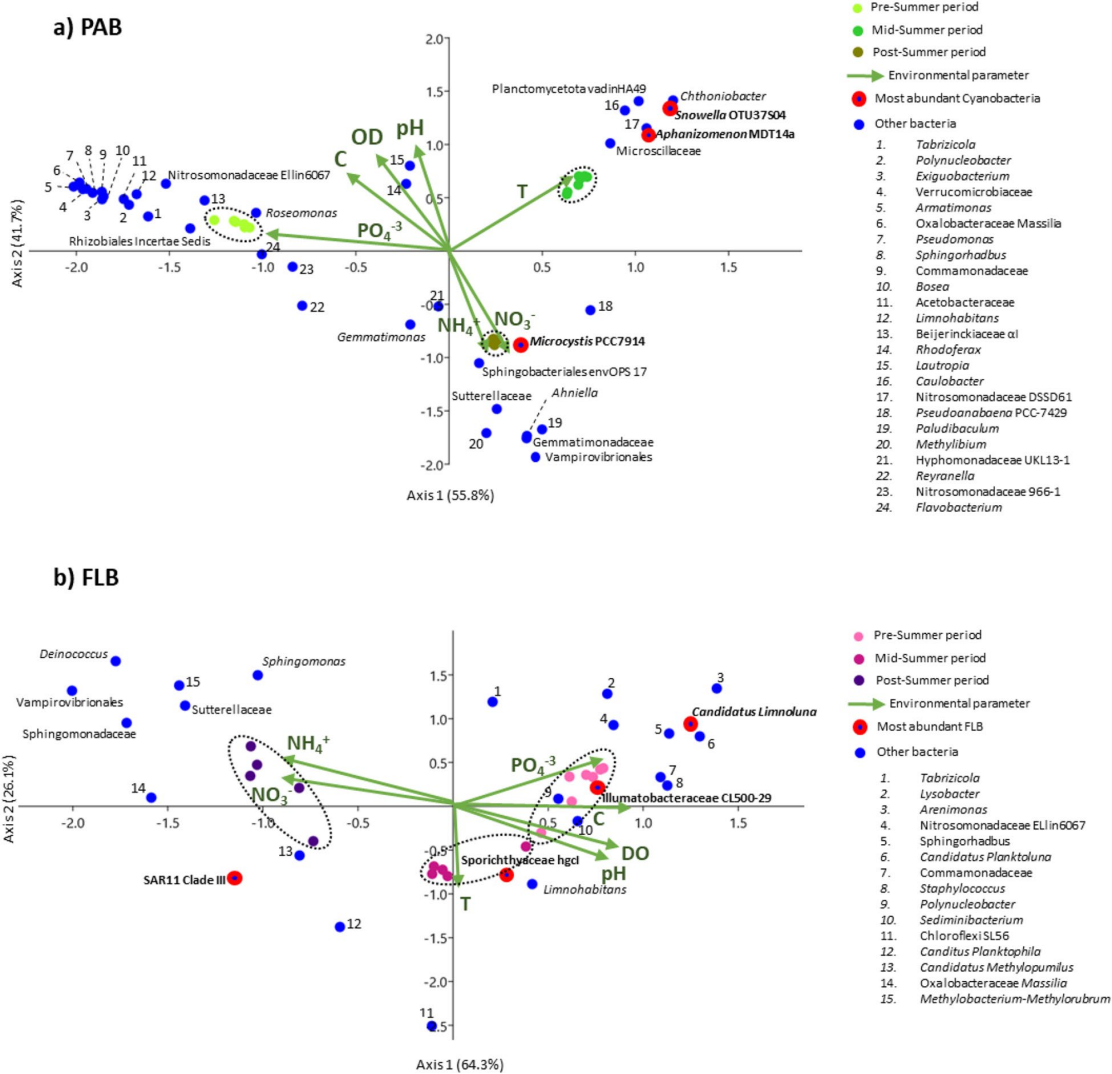
Canonical correspondence analysis (CCA) showing the relationships between bacterial community (OTUs) patterns and in situ environmental parameters in summer season. (**a**) particle-attached bacterial fraction (PAB), and (**b**) free-living bacterial fraction (FLB). Significant differences (*p* < 0.05) between sampling periods were tested with PERMANOVA.

The spatiotemporal variability of bacterioplankton assemblages (PAB *vs* FLB) and the influence of environmental parameters were analysed separately with canonical correspondence analysis (CCA) in Fig. 4. The CCA loadings were presented in supplementary Table S6. In the case of PAB, the CCA1 and CCA2 represented up to 97.5% of the total variance of the observations (55.8% and 41.7%, respectively; Fig. 4a). The samples were clustered into groups and significantly segregated according to the period of collection, which was supported by pairwise PERMANOVA (*F* = 243 and *p* = 0.003, see supplementary Table S7). The segregation of PRE sampling period was explained by the highest concentration of PO_4_^3−^ and abundance of OTUs belonging to *Roseomonas*, Nitrosomonadaceae_Ellin6067, Rhizobiales_Incertae_Sedis, and several other closely associated genera (see numbers 1 to 12 in Fig. 4a). The MID was explained by the highest increase in temperature, and abundances of cyanobacteria belonging to *Snowella*_OTU37S04 and *Aphanizomenon*_MDT14a, which were closely associated to the attached bacteria belonging to Microscillaceae, *Chthoniobacter*, and Planctomycetota_vadinHA49, among others (Fig. 4a). The POST was explained by the highest concentrations of NH_4_^+^ and NO_3_^−^, and abundance of *Microcystis*_PCC7914 with the associated bacteria belonging to Sphingobacteriales_env.OPS17, Sutterellaceae, *Ahniella*, Gemmatimonadaceae, and Vampirovibrionales, among others (Fig. 4a).

In the case of FLB, the CCA showed that the samples and OTUs were more dispersed when compared to PAB. The POST sampling period was characterized with the longest significant distance in comparison to PRE and MID, which was supported by pairwise PERMANOVA (*F* = 13.15 and *p* < 0.03, see supplementary Table S7). Despite the above, the PRE was again explained by the highest concentration of PO_4_^3−^, and the abundances of FLB belonging to *Candidatus_Limnoluna* and Ilumatobacteraceae_CL500-29, among others (Fig. 4b). The MID was explained by the highest temperature and abundances of Sporichthyaceae_hgcI and *Limnohabitans*, among others (Fig. 4b). Furthermore, the POST was explained by the highest concentrations of NH_4_^+^ and NO_3_^-^, and the abundance of SAR11_CladeIII, among others (Fig. 4b).

### Network interactions within bacterioplankton assemblages

**Figure 5:**
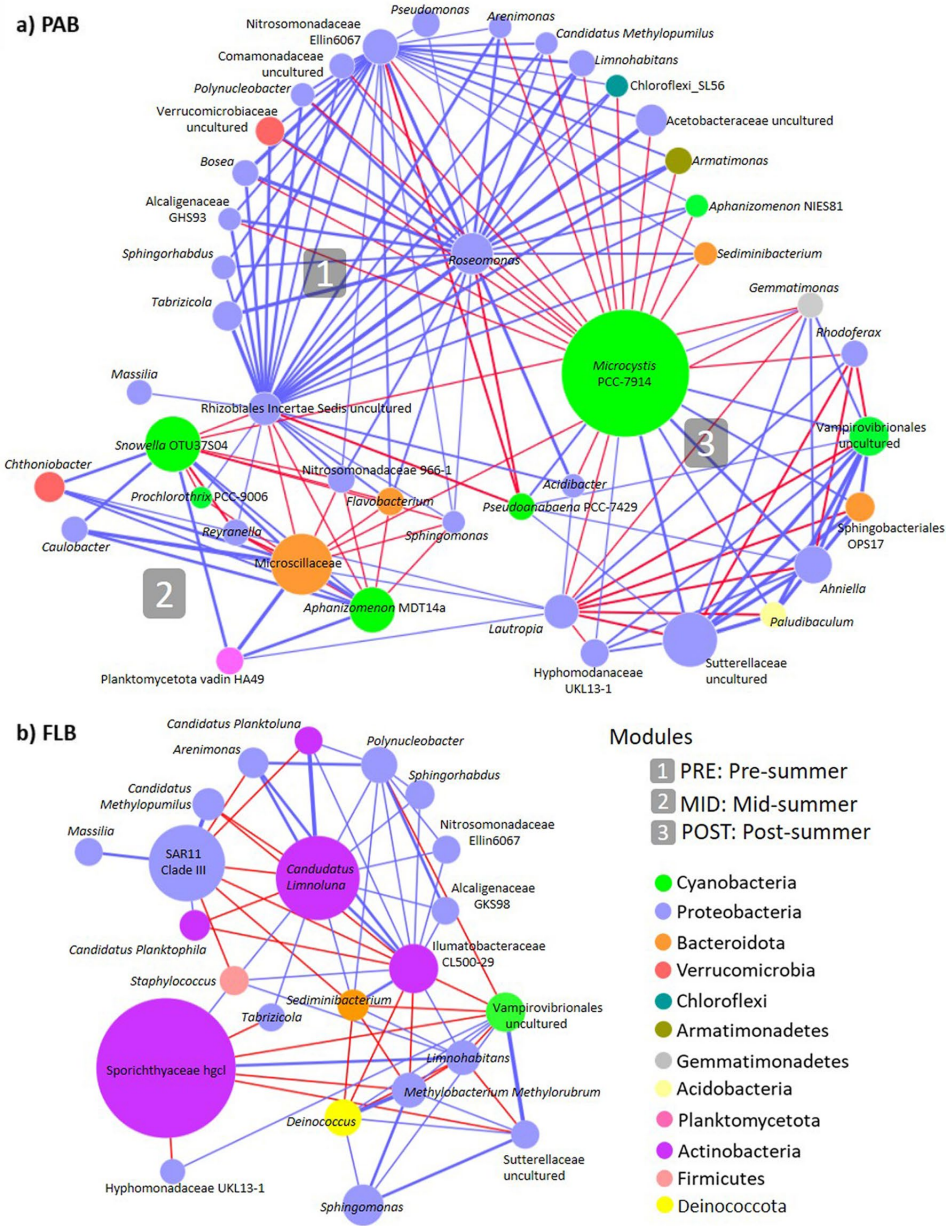
Network interactions between bacteria (OTUs) in PAB (**a**) and FLB (**b**). Only interactions with significant Spearman’s correlations where considered for the analysis (*p* < 0.05 and *r*_*s*_ > 0.6). Node size is proportional to the OTU average relative abundance, and the colours represent the phylum to which each OTU was assigned. Edge colours represent negative (red) and positive (blue) correlations, and the edge width represents the strength of the correlation. The network included OTUs that contributed with at least 1% of the average total abundance.

The co-occurrence network analysis was used to explore significant correlations among bloom-causing cyanobacteria and bacterioplankton assemblages. Therefore, two independent networks were constructed for PAB and FLB fractions respectively in Fig. 5, and the significant Spearman’s correlations between bacterial taxa (nodes and targets) were described in supplementary Tables S8 and S9. In the case of PAB network, a total of 158 significant correlations between bacterial OTUs were observed, from which positive relationships were more abundant than negative (108 over 54, respectively; Fig. 5a and Tables S8, S9). Furthermore, the network was also characterized with three modules representing the different sampling periods. The module 1 presented high abundant OTUs that were observed during PRE, with Rhizobiales_Incertae_Sedis, *Roseomonas*, and Nitrosomonadaceae_Ellin6067 as the most important nodes containing the highest number of positive relationships with other bacterial targets (23, 22, and 20, respectively; Fig. 5a and Tables S8, S9). The module 2 was represented by abundant OTUs during the MID, where the Bacteroidota belonging to Microscillaceae, and the cyanobacteria belonging to *Snowella*_OTU37S04 and *Aphanizomenon*_MDT14a, were the most abundant nodes containing higher numbers of positive relationships (5, 4, and 3, respectively; Fig. 5a and Tables S8, S9). Moreover, it is important to mention that these three OTUs were also observed to have a high number of negative relationships with other targets that were more abundant during PRE (7, 7, and 5, respectively; Fig. 5a and Tables S8, S9). The module 3 contained abundant taxa during POST, where *Microcystis*_PCC7914 was the most abundant node presenting the highest number of negative relationships (18) with targets that were more abundant during PRE (Fig. 5a and Tables S8, S9). Strong positive relationships between *Microcystis*_PCC7914 and other bacterial targets (8) were only observed in MID for *Pseudanabaena*_PCC7429 (*r*_*s*_ = 0.88, *p* = 2.03 × 10^–7^), and in POST for *Gemmatimonas* (*r*_*s*_ = 0.63, *p* = 2.43 × 10^–3^), Vampirovibrionales (*r*_*s*_ = 0.84, *p* = 2.33 × 10^–6^), Sphingobacteriales_env.OPS17 (*r*_*s*_ = 0.88, *p* = 1.05 × 10^–7^), *Paludibaculum* (*r*_*s*_ = 0.85, *p* = 1.10 × 10^–6^)*, Ahniella* (*r*_*s*_ = 0.92, *p* = 2.08 × 10^–9^), Sutterellaceae (*r*_*s*_ = 0.91, *p* = 6.96 × 10^–9^)*,* and Hyphomonadaceae_UKL13-1 (*r*_*s*_ = 0.62, *p* = 2.52 × 10^–3^) (Fig. 5a and Table S9)*.*

In the case of FLB, a total of 54 significant correlations were observed, where 43 were positive relationships and 11 were negative (Fig. 5b and Table S8). Modules were not easily distinguishable, and therefore, more difficult to discuss in terms of differences according to the sampling period. Despite the above, the Actinobacteriota was the most abundant phylum in FLB, with the highest number of significant correlations. The number of positive relationships were higher for Ilumatobacteraceae_CL500-29 and *Candidatus_Limnoluna* (10 and 9, respectively), while negative were higher for Ilumatobacteraceae_CL500-29 and Sporychthiaceae_hgcI (6 and 5, respectively) (Fig. 5b and Tables S8, S9).

## Discussion

### Temporal dynamics of cyanobacterial community

The present study illustrated the complex dynamic formation of bacterial communities in a reservoir periodically colonised by a toxigenic *Microcystis* bloom in the summer period, where the temperature and the concentration of nutrients were regarded as important physico-chemical factors contributing to such changes. The low temperature was probably an important factor inhibiting cyanobacterial development during PRE (16.8 °C; Fig. 1 and Table 1). Thereafter, the higher temperature in MID could have promoted their development (23.4 °C; Table 2 and Fig. 4a) with shared dominance among *Snowella*, *Microcystis*, and *Aphanizomenon* spp. (Fig. 1b). The lowest PO_4_^3-^ concentration could be the result of cyanobacterial uptake in the MID (23.6 µg L^−1^, Table 1), especially since blooms were observed abundant during that period. In previous studies, the bloom dynamic between *Aphanizomenon flos-aquae* and *Microcystis aeruginosa* was already described to occur at the Sulejów Reservoir^31,32^. Interestingly, *Snowella* 0TU37S04—closely associated to *S. litoralis*—was not known to occur in the Sulejów Reservoir. Despite the above, it is a common cyanobacterium found in central European lakes^33,34^, and it was also recently reported with parallel high abundance to *Microcystis* (27% and 62%, respectively) in mid-summer (August) at a reservoir in northern Germany^35^. Unfortunately, potential inter-relationships between both cyanobacterial communities were difficult to discuss due to the limited information on eco-physiological functions of *S. litoralis*. Further development of cyanobacterial bloom led to a significant dominance of *Microcystis* during the POST, with toxigenic genotypes (microcystin-producing gene *mcy*A) and microcystins production (3.87 µg L^−1^) (Fig. 2). Moreover, the lowest value in pH (7.2) and oxygen concentration (5.6 mg L^−1^) suggested that intensive microbial decomposition had started (Table 1), probably indicating that the POST bloom was already decaying (see “Post-summer period (POST)”). The highest concentrations of NH_4_^+^ and NO_3_^−^ (14.9 µg L^−1^ and 683 µg L^−1^, respectively) were regarded as important factors influencing towards *Microcystis* domination in POST (Fig. 4a), since they are known to be non-diazotrophic cyanobacteria that rely on external sources of N-compounds^36,37^. Previously, Mankiewicz et al.^38^ described *Microcystis* dynamics in the summer of five consecutive years at the Sulejów Reservoir, and concluded that high water retention, temperature, and optimum nutrient concentrations were key factors for the dominance of toxigenic microcystin-producing cyanobacteria. The highest concentration of dissolved inorganic nitrogen correlated with the highest abundances of toxigenic *Microcystis* at the summer of 2010, similarly as it was observed for the present study. In other studies, also nitrogen availability, rather than phosphorus, was regarded as one of the most important factors affecting the dominance of toxigenic over non-toxic populations of *Microcystis*, which in most cases is due to progressive anthropogenic eutrophication^39,40^.

### Temporal dynamics and potential eco-physiological function of particle-attached bacteria

#### Pre-summer period (PRE)

The lowest abundance of cyanobacteria was noticed at the beginning of summer (Fig. 1), which probably contributed to the highest diversity of associated bacterioplankton assemblages (apart from temperature and nutrient availability) (Table 1). These results suggested a higher number of habitats with many bacteria performing highly differentiated eco-physiological functions. The highest diversity and abundance of Proteobacteria corroborated the above observation, since they are known to occupy a broad variety of metabolic strategies that include the cycling of nutrients^41^. In the case of *Roseomonas,* many strains are known to contain the *nif*H gene involved in the process of nitrogen fixation^42^. In contrast, the Nitrosomonadaceae are well known to play an important role in the process of nitrification in freshwater ecosystems^43^, since they carry the genes involved in the process of ammonium oxidation to hydroxylamine (*amo*A) and subsequently to nitrite (*hao*)^44^. Furthermore, the Rhizobiales Incertae Sedis are uncultured bacteria only known from metagenomic analysis, however, they have been considered as important denitrifying communities since they were observed to quickly increase in abundance while removing high quantities of nitrate in waste water facilities^45^. The above observations suggested that they performed a key role in the recycling of nitrogen, and furthermore, they possibly were key elements acting synergistically with several other bacterioplankton during PRE (see Fig. 4a).

#### Mid-summer period (MID)

A significant decrease in the bacterioplankton diversity, evenness and richness were observed (Fig. 2), due to a strong dominance of cyanobacterial communities in the middle of summer (Fig. 1). The decrease in the number of significant relationships between bacterioplankton nodes for MID, when compared to the PRE, suggested that higher abundance of cyanobacteria had a negative impact on PAB. Although the MID cyanobacterial bloom was still not entirely dominated by toxic genotypes of *Microcystis* (Fig. 3), the high abundances of *Snowella* and *Aphanizomenon* spp. allowed the enrichment of bacterioplankton communities that were not found during other temporal sampling periods (Fig. 1c). The Microscillaceae has been linked as an obligate parasite of freshwater cyanobacteria^46^, and therefore, significant positive correlations observed between Microscillaceae and the cyanobacteria representing *Snowella* (*r*_*s*_ = 0.92, *p* = 2.03 × 10^–9^) and *Aphanizomenon* (*r*_*s*_ = 0.96, *p* = 1.11 × 10^–11^) in the present study suggested that they preyed upon these cyanobacterial communities (Fig. 5a). In a recent study, Chun et al.^47^ also distinguished Microscillaceae as a predatory agent of freshwater cyanobacteria, however, *Microcystis* was signalled as the main prey when both were in high concentrations during a bloom in Daechung Reservoir, Korea. Similarly, *Chthoniobacter* have been described to parasite freshwater filamentous cyanobacteria belonging to *Anabaena* and *Dolichospermum* during mixed blooms dominated by *Microcystis*^26,47^. In the present study, the abundance of Microscillaceae and *Chthoniobacter* were not correlated to *Microcystis*, probably indicating that there was a preference for the other two dominant cyanobacterial taxa (*r*_*s*_ = 0.95, *p* = 5.72 × 10^–11^ for *Snowella*, and *r*_*s*_ = 0.87, *p* = 3.73 × 10^–7^ for *Aphanizomenon,* see Fig. 5a). Finally, the significant increase of Planctomycetota (vadinHA49) also suggested its preference to *Snowella* (*r*_*s*_ = 0.91, *p* = 1.70 × 10^–8^) and *Aphanizomenon* (*r*_*s*_ = 0.95, *p* = 1.13 × 10^–10^) (Fig. 5a). They have been found previously abundant during intensive blooms^48,49^, with a preference to remain attached than free-living^50^. Furthermore, they are known to degrade complex polysaccharides, and therefore, it has been proposed that they feed on cyanobacterial exudates and decaying material^35,51^.

#### Post-summer period (POST)

A strong bloom dominated by *Microcystis* was probably an important factor reducing the PAB diversity and evenness in the POST (Fig. 2). The high abundance of negative strong correlations between *Microcystis* and several attached bacteria that were abundant during PRE, suggested that it was the cyanobacterium with the strongest negative impact to PAB communities in the summer of 2020 (Fig. 5a). The negative effect of *Microcystis* on attached bacterioplankton diversity has already been demonstrated in several previous studies^19,22,49,51^, and although its potential toxicity has been suggested to be an influential factor, it has not been extensively investigated. In the present study, the POST was significantly dominated by microcystin-producing genotypes (Fig. 3), indicating that this cyanotoxin could be an influential factor negatively affecting on PAB diversity. Microcystin has been described as an allelopathic compound inhibiting growth and photosynthesis in several phytoplankton species, including other cyanobacteria^52^, therefore implying that it may play a significant role in the dynamics of bacterioplankton communities. There are not many studies describing the potential role of toxic microcystin-producing genotypes on attached microorganisms. Scherer et al.^28^ investigated the temporal abundance of *mcy*B gene in two south German lakes, however bloom toxicity was not found to alter significantly the PAB. In contrast, Song et al.^53^ described an increase in PAB diversity parallel to high concentration of *mcy*ADH gene copy numbers and abundance of *Microcystis* during a summer bloom in Lake West, China. In both studies, toxic genotypes of *Microcystis* were present but not dominating the blooms, rather by a mixture of three or more cyanobacterial taxa, which could also explain the lack of negative effect on PAB as observed in the present study.

Potential eco-physiological functions of enriched PAB suggested that the POST bloom could have started the process of decaying. In the present study, the higher abundance of Sphingobacteriales (env.OPS 17) could be an indicator corroborating the above mentioned process (Fig. 1c), since the Sphingobacteriales order has already been described as a bloom specialist favouring the uptake of cyanobacterial exudates and decaying material^48,54^. Furthermore, this order has been proposed to contain microcystin-degrading bacteria due to its natural association with microcystin-producing cyanobacterial blooms^55^. In the case of the family env.OPS17, only two strains have been isolated from groundwater and are known to grow better on media containing bacterial cell lysate over simple carbon sources^56^. Such observation corroborates their preference on more complex carbon substrates, similarly as they could have encountered in a decaying environment dominated by *Microcystis* in the present study (Fig. 5a).

In the case of Sutterellaceae, it is known to naturally occur as gut microbiota, with representative strains isolated from animal and human faeces^57^. Its main origin could come from a variety of point and diffuse sources of pollution found above the Pilica River catchment and the Sulejów Reservoir, e.g.: domestic sewage and farming activities, respectively^58^, that could also explain its high abundance during POST (Fig. 1c). The above result portrayed Sutterellaceae as an important bioindicator of poor water quality during intensive blooms dominated by *Microcystis* in the present study. In the case of *Anhiella*, only one strain has been isolated from sandy soil near a stream in Sinan-gun, Korea^59^. Although its potential functional role is not known when associated to cyanobacterial blooms, the strain was able to utilize cellulose as a source of carbon, indicating that it also can use complex carbon substrates for growth. The above observations suggested that both, Sutterellaceae and *Anhiella*, could be opportunistic bacteria feeding on *Microcystis* exudates and decaying material. To our knowledge, the present study may represent the first report were both bacterial taxa were associated to blooms dominated by *Microcystis* (Fig. 5a).

High abundance of an uncultured strain of Vampirovibrionales was also associated to a parasitic lifestyle that could induce *Microcystis* cell lysis (Fig. 5a). However, parasitic lifestyle of this order was only demonstrated from the effects caused by one isolated strain—*Vampirovibrio chlorellavorus*—that is known to predate on the green algae *Chlorella* spp. Despite the above, Chun et al.^47^ described a positive correlation between Vampirovibrionales and *Microcystis* during a summer bloom in Daechung reservoir, Korea, suggesting that they could also predate on *Microcystis* cells. There are no other studies describing the above mentioned dynamics, and therefore, the results obtained in the present study could help to support the previous observation.

Potential nitrogen transforming bacteria belonging to *Roseomonas* and Nitrosomonadaceae (Ellin6067) were significantly depleted in the POST bloom (Fig. 1c). In contrast, these two bacterial taxa were described to be abundant during blooms dominated by *Microcystis*^48,51^. Furthermore, Qian et al.^60^ observed that abundances of nitrogen fixation (*nif*H) and denitrifying bacterial genes (*nir*SK, and *nos*Z) correlated to that of the microcystin-producing genes (*mcy*ADH) in Lake Tahoe, China, indicating that nitrogen transforming bacteria increased with concern to the appearance of toxic genotypes of *Microcystis* during a bloom in early August. Their results suggested that nitrogen transforming bacterial communities act synergistically with non-diazotrophic blooms, probably involved in the exchange of nitrogen compounds within the phycosphere of *Microcystis* cells. Therefore, a possible explanation on the antagonistic dynamics observed in the present study could be related on the decaying stage of *Microcystis* bloom during the POST (Fig. 5a). In another study, Chung et al.^47^ also observed that the abundance of *Roseomonas* significantly decreased in a post autumn bloom dominated by decaying *Microcystis*. Therefore, we speculated that microcystin could be a potential molecule affecting the development of these nitrogen transforming bacterial communities. In the case of polyphosphate accumulating bacteria, the *Gemmatimonas* has been proposed to facilitate the transfer of phosphorus to *Microcystis* cells in environments where the nutrient is depleted or in low concentrations^19,22^. In the present study, a strong correlation between both taxa suggested that *Gemmatimonas* provided an additional source of phosphorus for *Microcystis* in an environment limited with PO_4_^3−^ concentrations during the POST, therefore, playing a significant role to sustain *Microcystis* toxigenic blooms for longer periods of time.

### Temporal dynamics and potential eco-physiological function of free-living bacteria

FLB assemblages presented significant less connectivity between taxa (Fig. 5b), similarly as observed by Yang et al.^22^. This suggested that FLB ecological inter-relationships were not as complex as demonstrated for PAB assemblages (Fig. 5a). FLB were initially dominated by Actinobacteria (Fig. 1a), a phylum generally abundant in freshwater ecosystems^26,61^, however, independent from algal-derived compounds due to its ability to obtain supplementary light-derived energy from rhodopsins^19,62^. Its progressive decrease towards the POST indicated an antagonistic behaviour with the appearance of *Microcystis* dominated bloom (Fig. 1b), similarly as described in previous studies^26,63^. This behaviour was attributed to the increase in nutrient concentrations, that is usually followed by the appearance of blooms, considering that Actinobacteria prefer more oligotrophic conditions^28,62^. In the present study, we also observed that the concentration of NO_3_^-^ and NH_4_^+^ was the highest for the POST (Table 2 and Fig. 4b), which possibly contributed to a higher trophic condition resulting in a negative influence on Actinobacterial abundance. Interestingly, the significant Actinobacterial decrease allowed the enrichment of Proteobacteria at the POST (Fig. 1a), with SAR11_CladeIII as the most abundant genus during the strong bloom dominated by *Microcystis* (Fig. 1c). The SAR11_CladeIII was recently recognized as LD12 clade of the freshwater *Pelagibacter* to distinguish it from its close relatives in marine environments. Little is known about its eco-physiological function in the environment, however, genomic analysis suggested that it is composed of chemoorganotrophic bacteria feeding on simple organic compounds, and depending on sulphur, ammonia and other nitrogen sources^64^. Therefore, as it was mentioned before, the high concentration of nitrogen sources in the POST could have been an important factor influencing on its enrichment (Fig. 4b).

### Potential algicidal bacteria in attached consortia

Interestingly, several bacteria, e.g.: *Bacillus*, *Streptomyces*, *Pseudomonas*, *Aeromonas*, *Pseudoalteromonas*, *Alcaligenes*, *Lysinibacillus*, among others^65^, have been isolated with the ability to lyse *Microcystis aeruginosa* cells and other cyanobacterial species. However, none of them were observed in high abundance during the POST bloom dominated by *Microcystis* in the present study (Fig. 1c). One exception could be represented by the order Sphingobacteriales, that has been closely linked to algicidal activity^28^, and whose abundance in PAB during the POST bloom was high (Fig. 1c). In contrast, other studies have described the enrichment of the above mentioned algicidal bacteria during intense blooms^22,25,28,51^, although it is important to mention that the majority of known strains described in the literature are those culturable bacteria isolated by conventional methods; e.g. growing on nutrient-rich agar media^65^. Artificially culturable bacteria may represent less than 1% of the total diversity of bacteria, and therefore, we speculated that potential algicidal bacteria in the present study may be represented by species that are difficult to isolate and culture in laboratory conditions.

## Conclusions

Bacterioplankton communities were significantly different regarding to their structure, diversity, and inter-species relationships during the development of a toxigenic *Microcystis* bloom. The PAB, or phycosphere, showed more complex inter-relationships between cyanobacteria and other taxa, suggesting higher metabolic dependencies. Network analysis inferred that PRE was characterized with Proteobacterial potential taxa involved in nitrogen transformation processes, e.g.: nitrogen fixation, nitrification, and denitrification (*Roseomonas*, *Nitrosomonas*_Ellin6067 and Rhizobiales_incertae_sedis). PAB diversity decreased in MID and POST, proposing that cyanobacteria had a strong impact over bacterioplankton communities. Network analysis revealed that both periods were positively correlated to potential taxa known to have parasitic/predatory lifestyles (Microscillaceae and *Chthoniobacter*, and Vampirovibrionales) or were opportunistic towards the growth of cyanobacteria (Planctomycetota_vadinHA49, Sphingobacterales_env.OPS17, Sutterellaceae and *Anhiella*). The MID was composed with a mixed-bloom of *Snowella, Aphanizomenon*, and *Microcystis* that probably developed by the high registered temperature. The POST was significantly dominated by toxigenic *Microcystis* with production of MCs and the highest concentrations of NH_4_^+^ and NO_3_^−^ were regarded as important factors contributing to their domination. Toxigenic *Microcystis* was regarded as the taxon with the strongest negative impact on PAB due to numerous negative correlations (co-occurrences) registered with other bacterioplankton. In contrast, FLB presented less connectivity, suggesting no strong dependencies between taxa. They were mainly represented by Actinobacteria and Proteobacteria, and the highest concentration of nitrogen sources in the POST was regarded as an important factor influencing on Proteobacterial dominance. Such gathered knowledge may contribute to a better understanding in the deterioration of the ecological status of waters during blooms dominated by toxigenic *Microcystis*. This is essential to improve mitigation and contingency plans over development of CyanoHABs, for freshwater bodies that undergo increasing anthropogenic pressure.

## Methods

### Study site and collection of samples

The present study was performed in the Sulejów Reservoir, a freshwater lowland dammed reservoir located within the course of the Pilica River in Central Poland. Built with the purpose to provide flood control, recreation, power generation, and as an alternative source of water supply for the city of Lodz. The reservoir is under progressive anthropogenic eutrophication, with common appearance of toxigenic blooms dominated by *Microcystis aeruginosa* during the summer season^31,32,66–68^. Total cellular and free microcystin concentrations have reached up to 30 µg L^−1^ during intensive blooms^67^.

Sampling was performed in three consecutive periods during a bloom development in 2020: (i) the 04th of June representing the pre-summer period (PRE), (ii) the 13th of August as mid-summer (MID), and (iii) the 01st of October as post-summer (POST). For each sampling period, 5 L of surface water were collected and concentrated to 50 mL using a 20 µm phytoplankton net. Seven replicates were prepared for each sampling period. Particle-attached and free-living bacteria (PAB and FLB, respectively) were segregated according the specifications in Louati et al.^26^. Concentrated surface water samples were first filtered through 1.2 µm nitrocellulose filters (Millipore, USA) to collect the fraction containing PAB. Filtered water was then passed through 0.2 µm filters to collect the remaining FLB. Filters were stored at − 20 °C until further DNA extraction. Simultaneously, on the three above-mentioned days, samples were also taken for chemical and microcystins toxicity analyses.

### Physico-chemical parameters and chemical analysis of nutrients

Physico-chemical parameteres for surface water, temperature (T°), pH, oxygen concentration (mg L^−1^), and conductivity (µS cm^−1^), were measured in situ using a YSI multimetric probe. Filtrated water was used to estimate the concentration (mg L^−1^) of dissolved forms of P and N (PO_4_^3−^, NH_4_^+^, and NO_3_^−^) using Dionex ® ion chromatograph as specified by Gągała et al.^69^.

### Nucleic acid extraction, preparation of 16S rRNA libraries, and high-throughput sequencing

Filters containing PAB and FLB aggregates were placed into separate bead tubes and DNA was extracted according to the specifications in Dneasy ® PowerWater® Kit (Qiagen). Genomic DNA was measured with PicoGreen reagent (Life Technologies) using fluorimetry (Tecan’s Infinite). DNA libraries were prepared with the primers 341F and 785R targeting the region V3-V4 of the 16S rRNA gene^70^, with overhanging nucleotide adaptors recommended for Illumina technology^71^. The PCR reaction was performed using a Q5 Hot Start High-Fidelity 2 × Master Mix (New England Biolabs), with 3′ → 5′ exonuclease activity (~ 280-fold lower than that of *Taq* DNA polymerase), and the reaction conditions were followed as specified by the manufacturer. Sterile water was used as a negative control during PCR, and the DNA concentration was observed below threshold limit during the fluorometric measurement. The 16S rRNA V3-V4 amplicons were purified before attaching dual indices and Illumina sequencing adaptors according the specifications of Nextera XT Index kit^71^. Sequencing was performed in a MiSeq Illumina sequencer, using paired-end (PE) technology, 2 × 300 nt, with the MiSeq reagen kit v3 (600-cycle) according to the manufacturer specifications. DNA quantity, the preparation of 16S rRNA libraries, and sequencing were performed at the Genomed S.A. laboratories (http://www.genomed.pl/).

### Bioinformatic analysis

Reads were analysed with the QIIME2 version 2021.4 software package (https://qiime2.org/). The CUTEADAPT extension software was used to trim sequence adaptors and analyse the quality of the reading. Poor quality readings were removed (quality < 20, minimal length 30). The SEQPREP algorithm was applied to pair the sequences, and the USEARCH61 algorithm was used to remove chimeras. Finally, the UCLUST algorithm was used to cluster the sequences by similarity (97%) and assign a taxonomical unit (OTUs) based on the SILVA 138 classifier (https://www.arb-silva.de/). Sequences assigned to Chloroplast and Mitochondria were removed from the analysis.

### Quantitative analysis (qPCR)

PAB communities were also investigated with the use of real time qPCR. Two different markers were used to target the gene 16S rRNA to monitor the overall abundance of total bacteria and *Microcystis* spp. Furthermore, the gene *mcy*A was used to define the representative portion of the community that belonged to toxigenic *Microcystis* spp. The list of primers is presented in the Table 2. The reactions were performed using the Maxima SYBR Green/ROX master mix (Thermo Scientific) and the QuantStudio 3 real-time PCR system (Applied Biosystems). Each reaction (20 µL) was prepared in triplicate, containing 12.5 µL of ready to use master mix, 30 pmol of primers, and approximately 10 ng µL^−1^ of diluted DNA. The thermal cycling program included an initial denaturation at 94 °C for 10 min; 40 cycles of denaturation at 95 °C for 30 s, annealing for 30 s at different temperatures according to the genetic marker (Table 2), and extension at 72 °C for 30 s. A melting curve analysis was performed after qPCR with a reading ramp from 65 to 95 °C every 10 s, and the gene copy numbers were read at the phase of extension. A curve with six standards was prepared using genomic DNA isolated from a commercial strain of toxigenic *Microcystis aeruginosa* PCC7806. Calculations were made considering that 5,172,804 bp is the total size of the genome^72^. For all three qPCR assays, the efficiency of the reactions based on the standard curves were above 92%, with a high coefficient of determination (*r*^2^ > 0.99). The melting curve analysis showed defined sharp peaks for all three genes with no appearance of shoulder peaks (Supplementary material Fig. S2).

**Table 2:** Primers used for qPCR analysis in the present study.

Gene	Target	Primer	Sequence (5′ → 3′)	Annealing temp (°C)	Size (bp)
16S rRNA	Bacteria	BACT 1369f	CGGTGAATACGTTCYCGG	50	123^a^
		PROK 1492r	GGWTACCTTGTTACGACTT		
	*Microcystis* spp.	209F	ATGTGCCGCGAGGTGAAACCTAAT	55	250^b^
		409R	TTACAATCCAAAGACCTTCCTCCC		
*mcy*A	*Microcystis* spp. producing microcystin	mcyA-f1	AACCTATCCCGGTTGCTCAGATG	51	395^c^
		mcyA-r1	CACATCTCCAAGGAAAATACACCCC		

^a^Suzuki et al.^74^; ^b^Neilan et al.^75^; ^c^Gągała et al.^69^.

### Protein phosphatase inhibition assay

The protein phosphatase inhibition assay was performer with a MicroCystest screening immuno-bioassay (ZEU-Inmunotec, Zaragoza, Spain) to confirm the biological activity of microcystins (MCs), understood here as their toxicity. Collected samples containing cyanobacterial cells were prepared and analysed according to manufacturer’s instructions. MicroCystest is based on the inhibition of PP2A (rabbit skeletal muscle) activity by MCs, the mechanism of action used by these toxins in hepatocytes (PP2A is able to hydrolyse a specific substrate that can be detected at 405 nm). The test is able to detect all toxic MCs present in a sample. The assay range of the method is between 0.25 and 2.5 μg L^–1^. The concentration of MCs in the sample was expressed in microcystin-LR equivalents.

### Analysis of data

The diversity composition and multivariate ordination analyses were used to investigate differences between bacterioplankton assemblages (PAB and FLB) in three periods of collection (PRE, MID, and POST). The samples were first rarefied to estimate the sequencing depth, and then the α-diversity was described with the Simpson’s dominance (*D*), Shannon’s diversity (*H′*), Pielou’s eveness (*J*) and the Abundance-based coverage richness (ACE) indices. Differences in α-diversity between sampling periods were analysed with Kruskal–Wallis and Dunn’s post-hoc tests (*p* < 0.01). Furthermore, the canonical correspondence analysis (CCA) was used to describe relationships between bacterial OTUs (Genus) and in situ environmental parameters. Significant differences between community compositions through the sampling periods were evaluated with permutational multivariate analysis of variance (PERMANOVA, with 999 permutations) based on the Bray–Curtis dissimilarity index. Statistical analyses and visualization of CCA were performed with the software PAST 4.03^73^.

Co-occurrence network analysis was performed to investigate potential interactions between most significant OTUs (Genus) in PAB and FLB assemblages. The PAB network was performed with seven replicate samples for each period of collection, while the FLB network was performed with seven for PRE, and five for MID and POST respectively. Graphical visualization of networks were constructed with the software Cytoscape 3.8.2 (https://cytoscape.org/), focusing on interactions among different bacterial genera with strong (*r*_*s*_ > 6) and significant (*p* < 0.05) Spearman’s correlation. Furthermore, Spearman’s p-values were adjusted for multiple testing with Bonferroni correction. Topological properties were used to define networks in which a node represented an estimable species marker (OTUs), and the edge the correlation weight between two defined nodes. Furthermore, a positive or negative correlation between two nodes were interpreted as synergistic or antagonistic interactions respectively^51^.

## Supplementary Information

Supplementary Information.

## Data Availability

Original datasets as FASTQ files were uploaded to the NCBI Sequence Read Archive under the project PRJNA837559, with seven bio samples for the PREPAB: SAMN28206046–SAMN28206052, and seven for PREFLB: SAMN28206053–SAMN28206059; seven bio samples for the MIDPAB: SAMN28206060–SAMN28206066, and five for MIDFLB: SAMN28206067–SAMN28206071; seven bio samples for the POSTPAB: SAMN28206072–SAMN28206078, and five for POSTFLB: SAMN28206079–SAMN28206083.

## References

[1] Paerl HW (2018). Mitigating toxic planktonic cyanobacterial blooms in aquatic ecosystems facing increasing anthropogenic and climatic pressures. Toxins..

[2] Harke MJ, Steffen MM, Gobler CJ, Otten TG, Wilhelm SW, Wood SA (2016). A review of the global ecology, genomics, and biogeography of the toxic cyanobacterium *Microcystis* spp. Harmful Algae.

[3] Paerl HW, Barnard MA (2020). Mitigating the global expansion of harmful cyanobacterial blooms: Moving targets in a human- and climatically-altered world. Harmful Algae.

[4] Paerl HW (2014). Mitigating harmful cyanobacterial blooms in a human- and climatically-impacted world. Life..

[5] Burford MA, Carey CC, Hamilton DP, Huisman J, Paerl HW, Wood SA (2020). Perspective: Advancing the research agenda for improving understanding of cyanobacteria in a future of global change. Harmful Algae.

[6] Havens KE, James RT, East TL, Smith VH (2003). N: P ratios, light limitation, and cyanobacterial dominance in a subtropical lake impacted by non-point source nutrient pollution. Environ. Pollut..

[7] Bernard C (2014). Cyanobacteria and cyanotoxins. Rev. Franç. Lab..

[8] Paerl HW, Otten TG (2013). Harmful cyanobacterial blooms: Causes, consequences, and controls. Microb. Ecol..

[9] Dolman AM, Rücker J, Pick FR, Fastner J, Rohrlack T, Mischke U (2012). Cyanobacteria and cyanotoxins: The influence of nitrogen versus phosphorus. PLoS ONE.

[10] Svirčev Z, Lalić D, BojadžijaSavić G, Tokodi N, DrobacBacković D, Chen L (2019). Global geographical and historical overview of cyanotoxin distribution and cyanobacterial poisonings. Arch. Toxicol..

[11] Massey IY, Yang F (2020). A mini review on microcystins and bacterial degradation. Toxins.

[12] Paerl HW, Havens KE, Xu H, Zhu G, McCarthy MJ, Newell SE (2020). Mitigating eutrophication and toxic cyanobacterial blooms in large lakes: The evolution of a dual nutrient (N and P) reduction paradigm. Hydrobiologia.

[13] Sapp M, Schwaderer AS, Wiltshire KH, Hoppe HG, Gerdts G, Wichels A (2007). Species-specific bacterial communities in the phycosphere of microalgae?. Microb. Ecol..

[14] Cai H, Jiang H, Krumholz LR, Yang Z (2014). Bacterial community composition of size-fractioned aggregates within the phycosphere of cyanobacterial blooms in a eutrophic freshwater lake. PLoS ONE.

[15] Grant MAA, Kazamia E, Cicuta P, Smith AG (2014). Direct exchange of vitamin B 12 is demonstrated by modelling the growth dynamics of algal-bacterial cocultures. ISME J. Nat. Publ. Group.

[16] Shi L, Cai Y, Kong F, Yu Y (2012). Specific association between bacteria and buoyant Microcystis colonies compared with other bulk bacterial communities in the eutrophic Lake Taihu, China. Environ. Microbiol. Rep..

[17] Brunberg AK (1999). Contribution of bacteria in the mucilage of Microcystis spp (Cyanobacteria) to benthic and pelagic bacterial production in a hypereutrophic lake. FEMS Microbiol. Ecol..

[18] Shao K, Zhang L, Wang Y, Yao X, Tang X, Qin B (2014). The responses of the taxa composition of particle-attached bacterial community to the decomposition of *Microcystis* blooms. Sci. Total. Environ..

[19] Jankowiak JG, Gobler CJ (2020). The composition and function of microbiomes within microcystis colonies are significantly different than native bacterial assemblages in two North American lakes. Front. Microbiol..

[20] Bauer A, Forchhammer K (2021). Bacterial predation on cyanobacteria. Microb. Physiol..

[21] Ndlela LL, Oberholster PJ, Van Wyk JH, Cheng PH (2018). Bacteria as biological control agents of freshwater cyanobacteria: Is it feasible beyond the laboratory?. Appl. Microbiol. Biotechnol..

[22] Yang C, Wang Q, Simon PN, Liu J, Liu L, Dai X (2017). Distinct network interactions in particle-associated and free-living bacterial communities during a *Microcystis aeruginosa* bloom in a plateau lake. Front. Microbiol..

[23] Xu H, Zhao D, Huang R, Cao X, Zeng J, Yu Z (2018). Contrasting network features between free-living and particle-attached bacterial communities in Taihu Lake. Microb. Ecol..

[24] Liu M, Liu L, Chen H, Yu Z, Yang JR, Xue Y (2019). Community dynamics of free-living and particle-attached bacteria following a reservoir *Microcystis* bloom. Sci. Total Environ..

[25] Parveen B, Ravet V, Djediat C, Mary I, Quiblier C, Debroas D (2013). Bacterial communities associated with *Microcystis* colonies differ from free-living communities living in the same ecosystem. Environ. Microbiol. Rep..

[26] Louati I, Pascault N, Debroas D, Bernard C, Humbert JF, Leloup J (2015). Structural diversity of bacterial communities associated with bloom-forming freshwater cyanobacteria differs according to the cyanobacterial genus. PLoS ONE.

[27] Zwirglmaier K, Keiz K, Engel M, Geist J, Raeder U (2015). Seasonal and spatial patterns of microbial diversity along a trophic gradient in the interconnected lakes of the Osterseen Lake District, Bavaria. Front. Microbiol..

[28] Scherer PI, Millard AD, Miller A, Schoen R, Raeder U, Geist J (2017). Temporal dynamics of the microbial community composition with a focus on toxic cyanobacteria and toxin presence during harmful algal blooms in two South German lakes. Front. Microbiol..

[29] Kokocinski M, Dziga D, Antosiak A, Soininen J (2021). Are bacterio- and phytoplankton community compositions related in lakes differing in their cyanobacteria contribution and physico-chemical properties?. Genes.

[30] Dziga D, Kokociński M, Barylski J, Nowicki G, Maksylewicz A, Antosiak A (2019). Correlation between specific groups of heterotrophic bacteria and microcystin biodegradation in freshwater bodies of central Europe. FEMS Microbiol. Ecol..

[31] Jurczak T, Tarczynska M, Izydorczyk K, Mankiewicz J, Zalewski M, Meriluoto J (2005). Elimination of microcystins by water treatment processes: Examples from Sulejow Reservoir, Poland. Water Res..

[32] Mankiewicz-Boczek J, Izydorczyk K, Zdzisława R-D, Jurczak T, Karolina S, Kokocinski M (2006). Detection and monitoring toxigenicity of cyanobacteria by application of molecular methods. Environ Toxicol..

[33] Rajaniemi-Wacklin P, Rantala A, Mugnai MA, Turicchia S, Ventura S, Komárková J (2006). Correspondence between phylogeny and morphology of Snowella spp. and Woronichinia naegeliana, cyanobacteria commonly occurring in lakes. J. Phycol..

[34] DrobacBacković D, Tokodi N, Marinović Z, Lujić J, Dulić T, Simić SB (2021). Cyanobacteria, cyanotoxins, and their histopathological effects on fish tissues in Fehérvárcsurgó reservoir Hungary. Environ. Monit. Assess..

[35] Kallscheuer N, Rast P, Jogler M, Wiegand S, Kohn T, Boedeker C (2021). Analysis of bacterial communities in a municipal duck pond during a phytoplankton bloom and isolation of Anatilimnocola aggregata gen. nov., sp. Nov., Lacipirellula limnantheis sp. Nov. and Urbifossiella limnaea gen. nov. sp. nov. belonging to the phylum. Environ. Microbiol..

[36] Davis TW, Harke MJ, Marcoval MA, Goleski J, Orano-Dawson C, Berry DL (2010). Effects of nitrogenous compounds and phosphorus on the growth of toxic and non-toxic strains of Microcystis during cyanobacterial blooms. Aquat. Microb. Ecol..

[37] Gobler CJ, Davis TW, Coyne KJ, Boyer GL (2007). Interactive influences of nutrient loading, zooplankton grazing, and microcystin synthetase gene expression on cyanobacterial bloom dynamics in a eutrophic New York lake. Harmful Algae.

[38] Mankiewicz-Boczek J, Jaskulska A, Pawełczyk J, Gągała I, Serwecińska L, Dziadek J (2016). Cyanophages infection of microcystis bloom in lowland dam reservoir of Sulejów, Poland. Microb. Ecol..

[39] Davis TW, Berry DL, Boyer GL, Gobler CJ (2009). The effects of temperature and nutrients on the growth and dynamics of toxic and non-toxic strains of *Microcystis* during cyanobacteria blooms. Harmful Algae.

[40] Yoshida M, Yoshida T, Takashima Y, Hosoda N, Hiroishi S (2007). Dynamics of microcystin-producing and non-microcystin-producing *Microcystis* populations is correlated with nitrate concentration in a Japanese lake. FEMS Microbiol. Lett..

[41] Sezenna ML (2011). Proteobacteria: Phylogeny, Metabolic Diversity and Ecological Effects.

[42] Rilling JI, Acuña JJ, Sadowsky MJ, Jorquera MA (2018). Putative nitrogen-fixing bacteria associated with the rhizosphere and root endosphere of wheat plants grown in an andisol from southern Chile. Front. Microbiol..

[43] Lukumbuzya M, Kristensen JM, Kitzinger K, Pommerening-Röser A, Nielsen PH, Wagner M (2020). A refined set of rRNA-targeted oligonucleotide probes for in situ detection and quantification of ammonia-oxidizing bacteria. Water Res..

[44] Prosser JI, Head IM, Stein LY, Rosenberg E, DeLong EF, Lory S, Stackebrandt E, Thompson F (2014). The family Nitrosomonadaceae. The Prokaryotes: Alphaproteobacteria and Betaproteobacteria.

[45] Jia L, Jiang B, Huang F, Hu X (2019). Nitrogen removal mechanism and microbial community changes of bioaugmentation subsurface wastewater infiltration system. Bioresour. Technol..

[46] Daft MJ, Stewart WDP (1971). Bacterial pathogens of freshwater blue-green algae. New Phytol..

[47] Chun SJ, Cui Y, Lee JJ, Choi IC, Oh HM, Ahn CY (2020). Network analysis reveals succession of Microcystis genotypes accompanying distinctive microbial modules with recurrent patterns. Water Res..

[48] Parulekar NN, Kolekar P, Jenkins A, Kleiven S, Utkilen H, Johansen A (2017). Characterization of bacterial community associated with phytoplankton bloom in a eutrophic lake in South Norway using 16S rRNA gene amplicon sequence analysis. PLoS ONE.

[49] Guedes IA, Rachid CTCC, Rangel LM, Silva LHS, Bisch PM, Azevedo SMFO (2018). Close link between harmful cyanobacterial dominance and associated bacterioplankton in a tropical eutrophic reservoir. Front. Microbiol..

[50] Allgaier M, Grossart HP (2006). Seasonal dynamics and phylogenetic diversity of free-living and particle-associated bacterial communities in four lakes in northeastern Germany. Aquat. Microb. Ecol..

[51] Chen S, Yan M, Huang T, Zhang H, Liu K, Huang X (2020). Disentangling the drivers of *Microcystis* decomposition: Metabolic profile and co-occurrence of bacterial community. Sci. Total Environ..

[52] Leflaive J, Ten-Hage L (2007). Algal and cyanobacterial secondary metabolites in freshwaters: A comparison of allelopathic compounds and toxins. Freshw. Biol..

[53] Song H, Xu J, Lavoie M, Fan X, Liu G, Sun L (2017). Biological and chemical factors driving the temporal distribution of cyanobacteria and heterotrophic bacteria in a eutrophic lake (West Lake, China). Appl. Microbiol. Biotechnol..

[54] Bagatini IL, Eiler A, Bertilsson S, Klaveness D, Tessarolli LP, Vieira AAH (2014). Host-specificity and dynamics in bacterial communities associated with bloom-forming freshwater phytoplankton. PLoS ONE.

[55] Kohler E, Villiger J, Posch T, Derlon N, Shabarova T, Morgenroth E (2014). Biodegradation of microcystins during gravity-driven membrane (GDM) ultrafiltration. PLoS ONE.

[56] Wu X, Spencer S, Gushgari-Doyle S, Yee MO, Voriskova J, Li Y (2020). Culturing of “unculturable” subsurface microbes: Natural organic carbon source fuels the growth of diverse and distinct bacteria from groundwater. Front. Microbiol..

[57] Morotomi M, Nagai F, Watanabe Y (2011). Parasutterella secunda sp. no., isolated from human faeces and proposal of Sutterellaceae fam. nov. in the order Burkholderiales. Int. J. Syst. Evol. Microbiol..

[58] Kiedrzyńska E, Kiedrzyński M, Urbaniak M, Magnuszewski A, Skłodowski M, Wyrwicka A (2014). Point sources of nutrient pollution in the lowland river catchment in the context of the baltic Sea eutrophication. Ecol. Eng..

[59] Hwang WM, Ko Y, Kim JH, Kang K (2018). Ahniella affigens gen Nov, sp. nov., a gammaproteobacterium isolated from sandy soil near a stream. Int. J. Syst. Evol. Microbiol..

[60] Qian H, Lu T, Song H, Lavoie M, Xu J, Fan X (2017). Spatial variability of cyanobacteria and heterotrophic bacteria in Lake Taihu (China). Bull. Environ. Contam. Toxicol..

[61] Humbert JF, Dorigo U, Cecchi P, Le Berre B, Debroas D, Bouvy M (2009). Comparison of the structure and composition of bacterial communities from temperate and tropical freshwater ecosystems. Environ. Microbiol..

[62] Newton RJ, Jones SE, Eiler A, McMahon KD, Bertilsson S (2011). A guide to the natural history of freshwater lake Bacteria. Microbiol. Mol. Biol. Rev..

[63] Parveen B, Mary I, Vellet A, Ravet V, Debroas D (2013). Temporal dynamics and phylogenetic diversity of free-living and particle-associated *Verrucomicrobia* communities in relation to environmental variables in a mesotrophic lake. FEMS Microbiol. Ecol..

[64] Henson MW, Lanclos VC, Faircloth BC, Thrash JC (2018). Cultivation and genomics of the first freshwater SAR11 (LD12) isolate. ISME J..

[65] Yang C, Hou X, Wu D, Chang W, Zhang X, Dai X (2020). The characteristics and algicidal mechanisms of cyanobactericidal bacteria, a review. World J. Microbiol. Biotechnol..

[66] Izydorczyk K, Jurczak T, Wojtal-Frankiewicz A, Skowron A, Mankiewicz-Boczek J, Tarczyńska M (2008). Influence of abiotic and biotic factors on microcystin content in *Microcystis aeruginosa* cells in a eutrophic temperate reservoir. J. Plankton Res..

[67] Mankiewicz-Boczek J, Gągała I, Jurczak T, Jaskulska A, Pawełczyk J, Dziadek J (2015). Bacteria homologus to Aeromonas capable of microcystin degradation. Open Life Sci..

[68] Jaskulska A, Font Nájera A, Czarny P, Serwecińska L, Mankiewicz-boczek J (2021). Daily dynamic of transcripts abundance of Ma-LMM01-like cyanophages in two lowland European reservoirs. Ecohydrol. Hydrobiol..

[69] Gągała I, Izydorczyk K, Jurczak T, Pawełczyk J, Dziadek J, Wojtal-Frankiewicz A (2014). Role of environmental factors and toxic genotypes in the regulation of microcystins-producing cyanobacterial blooms. Microb. Ecol..

[70] Klindworth A, Pruesse E, Schweer T, Peplies J, Quast C, Horn M (2013). Evaluation of general 16S ribosomal RNA gene PCR primers for classical and next-generation sequencing-based diversity studies. Nucleic Acids Res..

[71] Illumina. *16S Metagenomic Sequencing Library Preparation*. (2013). http://support.illumina.com/content/dam/illumina-support/documents/documentation/chemistry_documentation/16s/16s-metagenomic-library-prep-guide-15044223-b.pdf.

[72] Frangeul L, Quillardet P, Castets AM, Humbert JF, Matthijs HCP, Cortez D (2008). Highly plastic genome of Microcystis aeruginosa PCC 7806, a ubiquitous toxic freshwater cyanobacterium. BMC Genomics.

[73] Hammer Ø, Harper DAT, Ryan PD (2001). Past: Paleontological statistics software package for education and data analysis even a cursory glance at the recent paleontological literature should convince anyone tha. Palaeontol. Electron..

[74] Suzuki MT, Taylor LT, DeLong EF (2000). Quantitative analysis of small-subunit rRNA genes in mixed microbial populations via 5’-nuclease assays. Appl. Environ. Microbiol..

[75] Neilan BA, Jacobs D, Del Dot T, Blackall LL, Hawkins PR, Cox PT, Goodman AE (2000). rRNA sequences and evolutionary relationships among toxic and nontoxic cyanobacteria of the genus. Microcystis. Int J Syst Bacteriol.

