# Supplementary material for: Temporal and functional interrelationships between bacterioplankton communities and the development of a toxigenic Microcystis bloom in a lowland European reservoir
Source: Sci Rep. 2022 Nov 11;12:19332. doi: 10.1038/s41598-022-23671-2 (PMC9652341; doi:10.1038/s41598-022-23671-2)
Supplement: Supplementary file 1 — Supplementary Information. [file 41598_2022_23671_MOESM1_ESM.pdf]

Temporal and functional interrelationships between bacterioplankton communities and the development of a toxigenic *Microcystis* bloom in a lowland European reservoir

Joanna Mankiewicz-Boczek[a]*, Arnoldo Font-Nájera[a]*

[a] European Regional Centre for Ecohydrology of the Polish Academy of Sciences, Tylna 3, 90-364 Łódź, Poland.

***Joanna Mankiewicz-Boczek** (Corresponding author)

e-mail: j.mankiewicz@erce.unesco.lodz.pl

3 Tylna, 90-364 Łódź, Poland

Tel: +48 (42) 681 70 07, Fax: +48 (42) 681 30 69

*Arnoldo Font-Nájera a.font-najera@erce.unesco.lodz.pl (Corresponding author)

SUPPLEMENTARY MATERIAL

In the present document, we summarize the different tables and graphs that are supplemented for the interpretation of results in the main manuscript.

Table S1. Summary of bioinformatic analysis for the sequencing of 16S rRNA gene (1/2)

Sample - Replicate	Raw reads	Passing QC		Pairs joined		Reads classified		Phylum		Order		Genera	
		Reads	%	Reads	%	Reads	%	Reads	%	Reads	%	Reads	%
PREPAB-01	107338	106597	99.31	89272	83.17	71956	67.04	71653	66.75	71642	66.74	69341	64.6
PREPAB-02	90942	90336	99.33	74240	81.63	55985	61.56	55802	61.36	55796	61.35	54334	59.75
PREPAB-03	91176	90549	99.31	75200	82.48	60735	66.61	60531	66.39	60527	66.38	58380	64.03
PREPAB-04	74744	74200	99.27	60616	81.1	47292	63.27	47018	62.91	47007	62.89	45385	60.72
PREPAB-05	94874	94329	99.43	79027	83.3	62495	65.87	62241	65.6	62231	65.59	60118	63.37
PREPAB-06	77558	77039	99.33	62640	80.77	50672	65.33	50481	65.09	50471	65.08	48743	62.85
PREPAB-07	85975	85458	99.4	71016	82.6	57746	67.17	57595	66.99	57590	66.98	55720	64.81
PREFLB-01	78790	78293	99.37	65547	83.19	56967	72.3	56607	71.85	56545	71.77	56132	71.24
PREFLB-02	104685	104073	99.42	88570	84.61	77307	73.85	77132	73.68	77105	73.65	76683	73.25
PREFLB-03	106549	105931	99.42	89306	83.82	81588	76.57	81434	76.43	81434	76.43	80884	75.91
PREFLB-04	97076	96417	99.32	81878	84.34	74453	76.7	74251	76.49	74251	76.49	73412	75.65
PREFLB-05	76964	76443	99.32	62742	81.52	53833	69.95	53492	69.5	53492	69.5	52878	68.7
PREFLB-06	72052	71524	99.27	58651	81.4	52205	72.45	52067	72.26	52067	72.26	49585	68.82
PREFLB-07	35780	35398	98.93	27128	75.82	24208	67.66	24181	67.58	24181	67.58	24078	67.29
MIDPAB-01	89505	88980	99.41	75005	83.8	61052	68.21	60919	68.06	60919	68.06	60107	67.15
MIDPAB-02	84495	83977	99.39	70834	83.83	58716	69.49	58624	69.38	58623	69.38	57772	68.37
MIDPAB-03	66067	65527	99.18	53506	80.99	43835	66.35	43751	66.22	43751	66.22	43057	65.17
MIDPAB-04	108679	107995	99.37	91572	84.26	75549	69.52	75387	69.37	75387	69.37	74015	68.1
MIDPAB-05	100474	99799	99.33	82642	82.25	67227	66.91	67060	66.74	67060	66.74	65934	65.62
MIDPAB-06	95352	94787	99.41	79346	83.21	64198	67.33	64095	67.22	64095	67.22	63023	66.1
MIDPAB-07	85051	84386	99.22	69113	81.26	55121	64.81	54989	64.65	54989	64.65	54122	63.63

QC: Quality control; PRE: Pre-summer period, MID: Mid-summer period, PAB: particle-attached bacteria, FLB: free-living bacteria.

Table S1. Summary of bioinformatic analysis for the sequencing of 16S rRNA gene (2/2)

Sample - Replicate	Raw reads	Passing QC		Pairs joined		Reads classified		Phylum		Order		Genus	
		Reads	%	Reads	%	Reads	%	Reads	%	Reads	%	Reads	%
MIDFLB-1	84618	84049	99.33	68602	81.07	52770	62.36	52587	62.15	52586	62.15	50462	59.64
MIDFLB-2	108726	108026	99.36	91039	83.73	71964	66.19	71797	66.03	71796	66.03	69887	64.28
MIDFLB-3	80075	79506	99.29	65085	81.28	52990	66.18	52888	66.05	52887	66.05	51744	64.62
MIDFLB-4	95801	95214	99.39	79616	83.11	64512	67.34	64358	67.18	64358	67.18	62925	65.68
MIDFLB-5	91248	90678	99.38	76247	83.56	69634	76.31	69347	76	69347	76	69085	75.71
POSTPAB-01	105104	104491	99.42	88503	84.21	71669	68.19	71387	67.92	71382	67.92	70264	66.85
POSTPAB-02	95671	94987	99.29	78238	81.78	61589	64.38	61362	64.14	61362	64.14	60412	63.15
POSTPAB-03	90112	89608	99.44	75109	83.35	60001	66.58	59802	66.36	59799	66.36	58896	65.36
POSTPAB-04	77749	77197	99.29	63403	81.56	51133	65.77	50921	65.49	50917	65.49	50128	64.47
POSTPAB-05	94953	94412	99.43	80400	84.67	67811	71.42	67569	71.16	67568	71.16	66635	70.18
POSTPAB-06	92747	92207	99.42	76870	82.88	61883	66.72	61707	66.53	61705	66.53	60928	65.69
POSTPAB-07	95569	95009	99.41	79770	83.47	65101	68.12	64905	67.91	64904	67.91	63918	66.88
POSTFLB-01	111726	111045	99.39	95209	85.22	67632	60.53	67085	60.04	67076	60.04	65182	58.34
POSTFLB-02	91929	91349	99.37	76492	83.21	64070	69.7	63810	69.41	63809	69.41	62806	68.32
POSTFLB-03	95665	95109	99.42	81095	84.77	57785	60.4	57450	60.05	57448	60.05	56438	59
POSTFLB-04	80911	80364	99.32	66787	82.54	46471	57.43	46136	57.02	46134	57.02	44812	55.38
POSTFLB-05	99000	98467	99.46	85636	86.5	74931	75.69	74498	75.25	74495	75.25	73356	74.1
**TOTAL**	3,415,730	3,393,756	99.4	2,835,952	83.0	2,315,086	67.8	2,306,919	67.5	2,306,736	67.5	2,261,581	66.2

QC: Quality control; MID: Mid-summer period, POST: Post-summer period, PAB: particle-attached bacteria, FLB: free-living bacteria. Samples MIDFLB-6, MIDFLB-7, POSTFLB-6, AND POSTFLB7 were removed from the analysis due to significant low quality readings (<90%).

Table S2. Average number of total OTUs and Reads obtained after 16S rRNA gene sequencing (1/2)

	**BAB**									**FLB**									
	**PRE**		**MID**		**POST**		**TOTAL**			**PRE**		**MID**		**POST**		**TOTAL**		
**Phylum**	OTUs	Reads	OTUs	Reads	OTUs	Reads	**OTUs**	**Reads**	**% Reads**	OTUs	Reads	OTUs	Reads	OTUs	Reads	**OTUs**	**Reads**	**% Reads**
**Gammaproteobacteria**	125	16514.0	117	7322.7	130	16917.6	372	40754.3	**23.9**	107	10558.6	129	9102.0	140	9913.3	376	29573.7857	**16.3**
**Alphaproteobacteria**	107	19799.6	86	4609.3	102	5613.3	295	30022.1	**17.6**	82	6842.4	99	9763.2	113	22341.4	294	38947.0238	**21.5**
**Cyanobacteria**	18	3588.9	16	24328.1	19	27732.7	53	55649.7	**32.6**	17	1680.9	18	8167.5	21	10619.1	56	20467.5	11.3
**Actinobacteriota**	66	5404.6	34	492.1	45	423.4	145	6320.1	3.7	61	33931.1	60	21878.7	70	10370.1	191	66179.9048	**36.6**
**Bacteroidota**	65	4134.1	53	10427.7	62	4159.1	180	18721.0	**11.0**	44	2796.6	56	5449.3	67	1527.4	167	9773.21429	5.4
Verrucomicrobiota	22	3494.0	19	3095.7	20	247.0	61	6836.7	4.0	17	359.9	20	1948.8	20	261.7	57	2570.30952	1.4
Planctomycetota	15	340.3	12	2111.7	13	531.1	40	2983.1	1.7	12	205.7	14	1177.1	13	344.0	39	1726.85714	1.0
Deinococcota	1	28.9	1	0.9	1	3.1	3	32.9	0.02	1	132.9	1	185.5	1	3710.9	3	4029.19048	2.2
Firmicutes	12	529.4	8	15.1	8	36.4	28	581.0	0.34	16	1763.9	15	506.4	17	27.6	48	2297.78571	1.3
Gemmatimonadota	2	359.6	2	200.9	2	1176.4	6	1736.9	1.02	1	37.1	2	132.8	2	509.1	5	679.02381	0.4
Chloroflexi	20	434.3	11	16.6	18	123.4	49	574.3	0.34	6	170.0	12	1375.4	19	256.9	37	1802.33333	1.0
Bdellovibrionota	9	102.9	9	276.3	9	798.0	27	1177.1	0.69	5	70.3	9	314.2	9	629.2	23	1013.66667	0.6
Acidobacteriota	18	175.6	14	112.9	18	1133.4	50	1421.9	0.83	7	136.4	15	58.1	19	216.6	41	411.142857	0.23
Armatimonadota	2	1497.4	2	17.3	2	13.3	6	1528.0	0.90	2	62.4	2	7.5	2	4.5	6	74.5238095	0.04
Myxococcota	7	24.4	9	515.9	9	357.9	25	898.1	0.53	6	37.0	9	99.3	9	64.3	24	200.619048	0.11
Spirochaetota	6	53.9	7	21.1	8	141.6	21	216.6	0.13	1	1.1	8	110.0	8	277.6	17	388.761905	0.21
Patescibacteria	8	115.3	2	18.6	12	10.7	22	144.6	0.08	10	187.1	7	72.1	14	52.6	31	311.857143	0.17
WPS-2	1	24.0	0	0.0	1	315.0	2	339.0	0.20	1	0.7	0	0.0	1	67.5	2	68.2142857	0.04
Desulfobacterota	22	98.9	11	7.7	22	145.7	55	252.3	0.15	0	0.0	11	2.3	22	37.0	33	39.2380952	0.02
Campilobacterota	2	0.9	1	0.1	2	13.4	5	14.4	0.01	2	15.3	2	1.5	2	152.9	6	169.714286	0.09
SAR324	1	1.6	1	39.0	1	45.9	3	86.4	0.05	1	1.4	1	28.5	1	26.3	3	56.2380952	0.03

The phylum Proteobacteria is displayed as both classes: ϒ-Proteobacteria and α-Proteobacteria. PRE: Pre-summer period, MID: Mid-summer period, POST: Post-summer period, PAB: particle-attached bacteria, FLB: free-living bacteria. Values represent the averages calculated from the different replicates obtained for each sample. Bold numbers were discussed in the manuscript.

Table S2. Average number of total OTUs and Reads obtained after 16S rRNA gene sequencing (2/2)

	**BAB**									**FLB**								
	**PRE**		**MID**		**POST**		**TOTAL**			**PRE**		**MID**		**POST**		**TOTAL**		
**Phylum**	OTUs	Reads	OTUs	Reads	OTUs	Reads	**OTUs**	**Reads**	**% Reads**	OTUs	Reads	OTUs	Reads	OTUs	Reads	**OTUs**	**Reads**	**% Reads**
Dependentiae	0	0.0	2	45.4	2	11.6	4	57.0	0.03	1	2.4	2	27.7	2	19.3	5	49.4285714	0.03
Nitrospirota	2	8.6	2	1.0	3	55.7	7	65.3	0.04	0	0.0	2	0.3	3	11.1	5	11.452381	0.006
Nitrospinota	1	19.4	1	0.4	1	19.0	3	38.9	0.02	1	2.6	1	0.1	1	5.2	3	7.80952381	0.004
MBNT15	1	8.4	1	0.3	1	14.7	3	23.4	0.01	0	0.0	1	0.0	1	4.1	2	4.16666667	0.002
Sva0485	1	4.3	0	0.0	1	10.4	2	14.7	0.01	0	0.0	0	0.0	1	1.7	1	1.73809524	0.0010
WOR-1	0	0.0	0	0.0	0	0.0	0	0.0	0	1	15.1	0	0.0	0	0.0	1	15.1428571	0.008
Latescibacterota	1	7.7	1	0.1	1	2.6	3	10.4	0.006	0	0.0	1	0.0	1	0.4	2	0.45238095	0.0003
Methylomirabilota	1	0.1	0	0.0	1	7.3	2	7.4	0.004	0	0.0	0	0.0	1	1.2	1	1.21428571	0.0007
NB1-j	1	5.0	0	0.0	1	1.4	2	6.4	0.004	0	0.0	0	0.0	1	0.2	1	0.23809524	0.0001
Elusimicrobiota	0	0.0	0	0.0	1	0.7	1	0.7	0.0004	0	0.0	1	0.2	1	5.8	2	5.95238095	0.003
Fusobacteriota	0	0.0	1	0.4	1	2.3	2	2.7	0.002	0	0.0	1	0.1	2	3.4	3	3.45238095	0.002
Sumerlaeota	1	1.1	1	0.1	1	2.0	3	3.3	0.002	0	0.0	1	0.0	1	0.3	2	0.35714286	0.0002
Margulisbacteria	1	0.1	0	0.0	1	1.3	2	1.4	0.001	0	0.0	0	0.0	1	1.0	1	1.04761905	0.0006
Fibrobacterota	0	0.0	0	0.0	1	1.4	1	1.4	0.001	0	0.0	0	0.0	1	0.9	1	0.9047619	0.0005
Caldisericota	1	0.3	0	0.0	1	1.1	2	1.4	0.001	0	0.0	0	0.0	1	0.2	1	0.19047619	0.0001
Abditibacteriota	0	0.0	0	0.0	0	0.0	0	0.0	0	1	1.6	0	0.0	0	0.0	1	1.57142857	0.0009
**TOTAL**	540	56,777.4	424	53,677.6	521	60,070.1	**1485**	170,525.143	100.0	403	59,012.6	500	60,408.8	588	61,464.7	**1491**	180,886.0	100.0

PRE: Pre-summer period, MID: Mid-summer period, POST: Post-summer period, PAB: particle-attached bacteria, FLB: free-living bacteria. Values represent the averages calculated from the different replicates obtained for each sample. Bold numbers were discussed in the manuscript.

Table S3. Average abundance (%) of most representative bacterial taxa in bacterioplankton communities during summer season (1/4)

PHYLUM												
	PAB						FLB					
	PRE		MID		POST		PRE		MID		POST	
Abundance range (%)	Phylum	%	Phylum	%	Phylum	%	Phylum	%	Phylum	%	Phylum	%
Major > 10%	Proteobacteria	62.5	Cyanobacteria	51.4	Cyanobacteria	47.9	Actinobacteriota	59.0	Actinobacteriota	45.9	Proteobacteria	62.3
			Proteobacteria	19.6	Proteobacteria	35.9	Proteobacteria	30.2	Proteobacteria	35.5	Actinobacteriota	22.5
			Bacteroidota	17.1								
Medium 5-10%	Actinobacteriota	9.3	Verrucomicrobiota	5.1	Bacteroidota	6.6			Bacteroidota	7.8	Deinococcota	8.1
	Cyanobacteria	8.0										
	Bacteroidota	7.1										
	Verrucomicrobiota	6.0										
Minor 1-5%	Armatimonadota	2.6	Planctomycetota	3.5	Acidobacteriota	2.1	Bacteroidota	4.9	Verrucomicrobiota	3.0	Others	1.9
			Others	1.3	Gemmatimonadota	1.9	Firmicutes	3.1	Chloroflexi	2.9	Bacteroidota	1.8
					Bdellovibrionota	1.3	Verrucomicrobiota	0.6	Planctomycetota	1.7	Bdellovibrionota	1.1
							Planctomycetota	0.4	Firmicutes	1.1		
Lower < 1%	Others	4.5	Others	2.0	Others	4.4	Others	1.9	Others	2.1	Others	2.4
Total		100.0		100.0		100.0		100.0		100.0		100.0

PAB: particle-attached bacteria, FLB: free living bacteria. PRE: Pre-summer period, MID: Mid-summer period, POST: Post-summer period

ORDER												
	PAB						FLB					
	PRE		MID		POST		PRE		MID		POST	
Abundance range (%)	Order	%	Order	%	Order	%	Order	%	Order	%	Order	%
Major > 10%	Burkholderiales	23.2	Cyanobacteriales	43.7	Cyanobacteriales	39.1	Micrococcales	27.8	Frankiales	33.7	SAR11_clade	20.1
	Acetobacterales	11.2	Cytophagales	17.3	Burkholderiales	20.4	Frankiales	20.9	Burkholderiales	12.6	Frankiales	13.7
	Rhizobiales	11.2	Burkholderiales	11.1			Burkholderiales	12.0	SAR11_clade	10.9	Burkholderiales	12.1

Table S3. Average abundance (%) of most representative bacterial taxa in bacterioplankton communities during summer season (2/4)

	ORDER											
	PAB						FLB					
	PRE		MID		POST		PRE		MID		POST	
Abundance range (%)	Order	%	Order	%	Order	%	Order	%	Order	%	Order	%
Medium 5-10%	Cyanobacteriales	6.1	Chthoniobacterales	4.6	Vampirovibrionales	9.1	Microtrichales	7.5	Cyanobacteriales	6.6	Vampirovibrionales	9.3
					Xanthomonadales	8.2					Sphingomonadales	8.8
											Deinococcales	7.2
											Rhizobiales	7.0
Minor 1-5%	Rhodobacterales	4.2	Caulobacterales	3.4	Sphingobacteriales	2.9	Sphingomonadales	4.7	Microtrichales	4.2	Cyanobacteriales	2.2
	Verrucomicrobiales	4.2	Planctomycetota_vadinHA49	2.7	Cytophagales	2.7	Xanthomonadales	3.8	Micrococcales	3.4	Micrococcales	2.1
	Frankiales	3.9	Acetobacterales	2.3	Rhizobiales	2.7	Chitinophagales	2.6	Cytophagales	3.0	Microtrichales	2.1
	Pseudomonadales	3.2	Xanthomonadales	1.7	Caulobacterales	2.5	Synechococcales	2.0	Chloroflexi_SL56	2.6	Rickettsiales	1.4
	Armatimonadales	2.6	Rhizobiales	1.5	Gemmatimonadales	2.1	Staphylococcales	1.8	Rhizobiales	2.4	Caulobacterales	1.3
	Sphingomonadales	2.6	Pseudanabaenales	1.1	Acetobacterales	2.0	Rhizobiales	1.7	Sphingobacteriales	1.6	Rhodobacterales	1.2
	Flavobacteriales	2.5			Acidobacteriae	1.8	Rhodobacterales	1.7	Chitinophagales	1.6	Corynebacteriales	1.1
	Caulobacterales	2.4			Pseudanabaenales	1.5	Acetobacterales	1.2	Chthoniobacterales	1.4		
	Microtrichales	2.0			Silvanigrellales	1.1	Sphingobacteriales	1.0	Sphingomonadales	1.3		
	Cytophagales	2.0			Reyranellales	1.1	Pseudomonadales	1.0	Synechococcales	1.2		
	Micrococcales	1.9			Flavobacteriales	1.1			Incertae_Sedis	1.0		
	Chitinophagales	1.5							Caulobacterales	1.0		
	Rickettsiales	1.3										
	Corynebacteriales	1.3										
	Reyranellales	1.2										
	Sphingobacteriales	1.0										
Lower <1%	Others	10.4	Others	10.7	Others	10.3	Others	10.3	Others	11.7	Others	10.5
Total		100.0		100.0		108.7		100.0		100.0		100.0

PAB: particle-attached bacteria, FLB: free-living bacteria. PRE: Pre-summer period, MID: Mid-summer period, POST: Post-summer period.

Table S3. Average abundance (%) of most representative bacterial taxa in bacterioplankton communities during summer season (3/4)

GENUS												
	**PAB - w**ithout photosynthetic cyanobacteria						**FLB**					
	**PRE**		**MID**		**POST**		**PRE**		**MID**		**POST**	
Abundance range (%)	Genus	%	Genus	%	Genus	%	Genus	%	Genus	%	Genus	%
Major >10%			Microscillaceae_ uncultured	31.0	Sutterellaceae_uncultured	21.1	*Candidatus_Limnoluna*	24.9	Sporichthyaceae_hgcI	34.0	SAR11_Clade_III	20.6
					Vampirovibrionales_uncultured	13.5	Sporichthyaceae_hgcI	21.2	SAR11_Clade_III	11.8	Sporichthyaceae_hgcI	12.1
					Ahniella	11.2						
Medium 5-10%	*Roseomonas*	7.1	*Chthoniobacter*	8.2			Ilumatobacteraceae_ CL500-29	7.6	Ilumatobacteraceae_ CL500-29	4.3	Vampirovibrionales_ uncultured	9.5
	Nitrosomonadaceae_Ellin6067	6.2	*Lautropia*	6.0							*Sphingomonas*	7.5
											Deinococcus	7.3
											Methylobacterium-Methylorubrum	5.5
Minor 1-5%	Acetobacteraceae_uncultured	4.8	Planctomycetota_ vadinHA49	4.9	Sphingobacteriales_env.OPS_17	4.0	*Polynucleobacter*	3.3	*Limnohabitans*	3.5	*Candidatus_ Methylopumilus*	2.6
	Rhizobiales_Incertae_Sedis_ uncultured	4.6	*Caulobacter*	3.9	Microscillaceae_uncultured	3.9	*Arenimonas*	3.0	*Candidatus_Limnoluna*	3.0	*Massilia*	2.2
	Tabrizicola	3.7	*Roseomonas*	3.7	*Paludibaculum*	2.7	*Sphingomonas*	2.2	Microscillaceae_ uncultured	2.9	Sutterellaceae_uncultured	2.1
	Verrucomicrobiaceae_uncultured	3.7	Sutterellaceae_uncultured	2.6	Hyphomonadaceae_UKL13-1	2.5	*Limnohabitans*	2.1	Chloroflexi_SL56	2.8	Ilumatobacteraceae_ CL500-29	1.6
	Sporichthyaceae_hgcI	3.2	*Rhodoferax*	2.1	*Roseomonas*	2.5	*Sphingorhabdus*	2.0	*Candidatus_ Planktophila*	2.4	*Candidatus_Planktophila*	1.5
	Pseudomonas	3.1	Nitrosomonadaceae_ Ellin6067	1.8	*Gemmatimonas*	1.6	*Sediminibacterium*	2.0	*Polynucleobacter*	2.1	*Candidatus_Limnoluna*	1.5
	Lautropia	2.8	Hyphomonadaceae_ UKL13-1	1.4	*Reyranella*	1.6	*Staphylococcus*	1.8	*Candidatus_ Methylopumilus*	1.9	*Polynucleobacter*	1.3
	Armatimonas	2.8	Sphingobacteriales_ env.OPS_17	1.1	Rhizobiales_Incertae_Sedis_ uncultured	1.5	*Candidatus_ Planktoluna*	1.6	*Sediminibacterium*	1.6		
	Limnohabitans	2.4	Sporichthyaceae_hgcI	1.0	*Flavobacterium*	1.3	Alcaligenaceae_GKS98	1.4	*Acidibacter*	1.1		
	Bosea	2.3	*Ahniella*	1.0	Nitrosomonadaceae_966-1	1.3	Nitrosomonadaceae_Ellin6067	1.4				
	Flavobacterium	2.1					*Tabrizicola*	1.2				
	Massilia	1.8					*Roseomonas*	1.0				
	Microscillaceae_uncultured	1.7										
	Sutterellaceae_uncultured	1.7										
	Comamonadaceae_uncultured	1.7										

BAB: particle-attached bacteria, FLB: free-living bacteria. PRE: Pre-summer period, MID: Mid-summer period, POST: Post-summer period.

Table S3. Average abundance (%) of most representative bacterial taxa in bacterioplankton communities during summer season (4/4)

GENUS												
	**PAB -** Without photosynthetic cyanobacteria						**FLB**					
	**PRE**		**MID**		**POST**		**PRE**		**MID**		**POST**	
Abundance range (%)	Genus	%	Genus	%	Genus	%	Genus	%	Genus	%	Genus	%
Minor 1-5%	Nitrosomonadaceae_966-1	1.5										
	Ilumatobacteraceae_CL500-29	1.4										
	Reyranella	1.3										
	Candidatus_Limnoluna	1.2										
	Sphingorhabdus	1.2										
	Rhodoferax	1.1										
	Polynucleobacter	1.0										
Lower <1%	Others	35.6	Others	31.4	Others	31.2	Others	23.4	Others	28.7	Others	24.6
Total		100.0		100.0		100.0		100.0		100.0		100.0

BAB: particle-attached bacteria, FLB: free-living bacteria. PRE: Pre-summer period, MID: Mid-summer period, POST: Post-summer period.

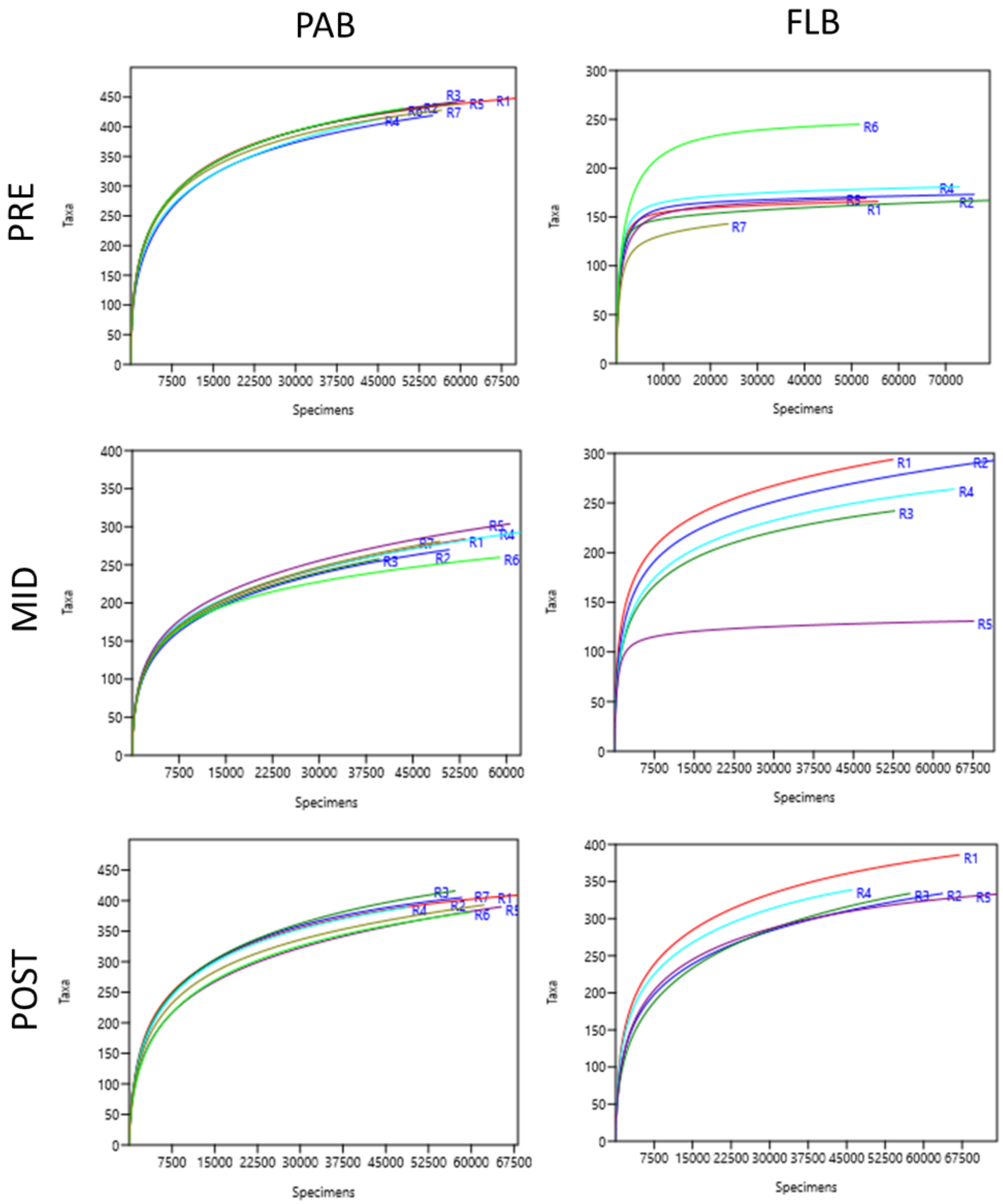

Fig S1. Individual rarefaction curves estimating the number of expected taxa according to the size of the sample. PAB: Particle-attached bacteria, FLB: free-living bacteria. PRE: Pre-summer period, MID: Mid-summer period, POST: Post-summer period. R1-R7: sample replicates.

Table S4. Statistical Kruskal Wallis (a) and Dunn's post-hoc (b) analyses to test significant differences between α-diversity indexes measured for bacterioplankton assemblages.

(a) Kruskal Wallis test

Parameter	PAB		FLB	
	Dominance (n = 21)	Shannon (n = 21)	Dominance (n = 17)	Shannon (n = 17)
H (chi^2):	16.48	13.45	2.178	0.4034
Hc (tie corrected):	16.48	13.45	2.178	0.4034
p (same):	2.64E-04	1.20E-03	0.3365	0.8174
Conclusion	There is a significant difference between sample medians, Dunn's post-hoc test was performed to observe specific differences between samples		There is no significant difference between sample medians	

Significant values are marked in red colour ($p < 0.01$).

Parameter	PAB		FLB	
	Pielou's evenness (n = 21)	Abundance-based coverage (n = 21)	Pielou's evenness (n = 17)	Abundance-based coverage (n = 17)
H (chi^2):	16.79	16.19	2.259	11.48
Hc (tie corrected):	16.79	16.19	2.259	11.48
p (same):	2.26E-04	3.06E-04	0.3232	3.21E-03
Conclusion	There is a significant difference between sample medians, Dunn's post-hoc test was performed to observe specific differences between samples		There is no significant difference between sample medians for Pielou's evenness index. There is a significant difference between sample medians for abundance-based coverage index.	

Significant values are marked in red colour ($p < 0.01$).

(b) Dunn's post-hoc test

Dominance index (*D*) for PAB assemblages

	Mid-AB	Post-AB
Pre-AB	0.06731	1.54×10^{-4}
Mid-AB		0.2322

Significant values are marked in red colour after Bonferroni correction ($p < 0.01$).

Shannon index (*H'*) for PAB assemblages

	Mid-AB	Post-AB
Pre-AB	7.71×10^{-3}	2.73×10^{-3}
Mid-AB		0.7015

Significant values are marked in red colour after Bonferroni correction ($p < 0.01$).

Pielou's evenness (J) for PAB assemblages

	Mid-AB	Post-AB
Pre-AB	0.04531	1.28×10^{-4}
Mid-AB		0.192

Significant values are marked in red colour after Bonferroni correction ($p < 0.01$).

Abundance-based coverage (ACE) for PAB assemblages

	Mid-AB	Post-AB
Pre-AB	1.85E^{-4}	0.279
Mid-AB		0.060

Significant values are marked in red colour after Bonferroni correction ($p < 0.01$).

Abundance-based coverage (ACE) for FLB assemblages

	Mid-AB	Post-AB
Pre-AB	0.6702	2.16E^{-3}
Mid-AB		0.135

Significant values are marked in red colour after Bonferroni correction ($p < 0.01$).

Table S5. Summary of gene copy numbers per mL (g# mL^{-1}) in BAB assemblages

	16S rRNA total bacteria (16S tb) g# mL^{-1}	16S rRNA *Microcystis* spp. (16S M) g# mL^{-1}	*mcy*A *Microcystis* spp. (*mcy*A M) g# mL^{-1}	16S tb / 16S M	16S M / *mcy*A M
PRE	$7.60 \times 10^{3} \pm 3.85 \times 10^{2}$	$9.51 \times 10^{1} \pm 0.45 \times 10^{1}$	$1.43 \times 10^{1} \pm 0.14 \times 10^{1}$	80.0	6.7
MID	$2.55 \times 10^{5} \pm 1.80 \times 10^{4}$	$9.22 \times 10^{3} \pm 5.60 \times 10^{2}$	$5.39 \times 10^{3} \pm 1.50 \times 10^{1}$	27.6	1.7
POST	$1.34 \times 10^{6} \pm 4.42 \times 10^{4}$	$1.42 \times 10^{5} \pm 1.09 \times 10^{4}$	$1.27 \times 10^{5} \pm 2.76 \times 10^{3}$	9.4	1.1

16S tb / 16S M value represents the size of total bacteria over total *Microcystis* spp. In turn, the 16S M / *mcy*A M value represents the size of total *Microcystis* spp. over toxigenic strains of *Microcystis* spp.

Table S6. CCA scores for bacterioplankton assemblages (1/2).

BAB		
**Parameter**	**CCA1**	**CCA2**
**Environmental parameter**		
Oxygen (mg L^{-1})	-0.409281	0.914341
pH	-0.205388	0.971551
Temperature (C°)	0.649581	0.748297
Conductivity (µS cm^{-1})	-0.643191	0.7212
NH_4^+	0.268411	-0.958907
NO_3^-	0.305831	-0.946554
NO_2^-	0.202055	-0.052967
PO_4^{3-}	-0.992584	0.113152
**Sample - replicate**		
PREBAB1	-1.13097	0.214551
PREBAB2	-1.10627	0.197285
PREBAB3	-1.19408	0.253468
PREBAB4	-1.13784	0.227038
PREBAB5	-1.18281	0.241879
PREBAB6	-1.13269	0.19754
PREBAB7	-1.30037	0.251481
MIDBAB1	0.691408	0.778037
MIDBAB2	0.670422	0.754857
MIDBAB3	0.705873	0.77082
MIDBAB4	0.660355	0.774598
MIDBAB5	0.602263	0.608437
MIDBAB6	0.6047	0.630825
MIDBAB7	0.661016	0.695873
POSTBAB1	0.272702	-0.821781
POSTBAB2	0.272556	-0.864986
POSTBAB3	0.280149	-0.821895
POSTBAB4	0.290739	-0.818482
POSTBAB5	0.27869	-0.835554
POSTBAB6	0.295465	-0.79055
POSTBAB7	0.279096	-0.826641
**OTU**		
Paludibaculum	0.553177	-1.51443
Armatimonas	-2.07583	0.504561
Microscillaceae_uncultured	0.819542	1.08394
Flavobacterium	-1.03034	-0.0388289
Sphingobacteriales_env.OPS_17	0.187519	-0.939812
*Microcystis*_PCC-7914	0.405194	-0.756548
*Snowella*_0TU37S04	1.13167	1.42721

FLB		
**Parameter**	**CCA1**	**CCA2**
**Environmental parameter**		
Oxygen (mg L^{-1})	0.926478	-0.31309
pH	0.825477	-0.512672
Temperature (C°)	0.0195074	-0.887796
Conductivity (µS cm^{-1})	0.953675	0.00542991
NH_4^+	-0.867628	0.437788
NO_3^-	-0.874997	0.39318
NO_2^-	0.0130008	-0.11975
PO_4^{3-}	0.619564	0.658147
**Sample - replicate**		
PREFLB1	0.614593	0.33718
PREFLB2	0.778168	0.429213
PREFLB3	0.622154	0.0496776
PREFLB4	0.702166	0.369456
PREFLB5	0.795836	0.433526
PREFLB6	0.741274	0.347066
PREFLB7	0.457672	-0.342849
MIDFLB1	-0.0594664	-0.789044
MIDFLB2	-0.105599	-0.700684
MIDFLB3	-0.034684	-0.805404
MIDFLB4	-0.115455	-0.760733
MIDFLB5	0.376113	-0.504306
POSTFLB1	-0.8063	0.224201
POSTFLB2	-1.05663	0.594572
POSTFLB3	-1.0268	0.388617
POSTFLB4	-1.06423	0.347899
POSTFLB5	-0.739886	-0.193623
**OTU**		
Ilumatobacteraceae_CL500-29	0.763409	0.125936
Candidatus_Planktophila	-0.59814	-1.42649
Sporichthyaceae_hgcI	0.26625	-0.851547
Candidatus_Limnoluna	1.24828	1.04492
Candidatus_Planktoluna	1.29425	1.10965
Sediminibacterium	0.657351	-0.230644
Microscillaceae_uncultured	-0.174836	-3.06883
Chloroflexi_SL56	-0.117468	-2.74353
Vampirovibrionales_uncultured	-1.9943	1.31515
Deinococcus	-1.76283	1.10768
Staphylococcus	1.107	0.180936

BAB: bloom attached bacteria; FLB: free living bacteria

Table S6. CCA scores for bacterioplankton assemblages (2/2).

BAB		
**Parameter**	**CCA1**	**CCA2**
**OTU**		
*Aphanizomenon*_MDT14a	1.02391	1.17724
*Pseudanabaena*_PCC-7429	0.772063	-0.423987
Exiguobacterium	-1.91614	0.397695
Gemmatimonas	-0.199353	-0.617945
Gemmatimonadaceae_ uncultured	0.472318	-1.59981
Planctomycetota_vadinHA49	0.958295	1.47327
Roseomonas	-1.07662	0.333311
Acetobacteraceae_uncultured	-1.79911	0.409675
Caulobacter	0.887071	1.38479
Hyphomonadaceae_UKL13-1	-0.0533831	-0.445075
Reyranella	-0.792668	-0.485823
Bosea	-1.91015	0.425719
Beijerinckiaceae_alphaI	-1.36026	0.426516
Rhizobiales_Incertae_Sedis_ uncultured	-1.42813	0.169888
Tabrizicola	-1.66055	0.262716
Sphingorhabdus	-2.02479	0.485491
Lautropia	-0.259117	0.810704
Polynucleobacter	-1.92072	0.467794
Limnohabitans	-1.76857	0.360121
Methylibium	0.252296	-1.56607
Rhodoferax	-0.273191	0.647022
Commamonadaceae_uncultured	-1.73159	0.45913
Nitrosomonadaceae_966-1	-0.858933	-0.139301
Nitrosomonadaceae_DSSD61	1.01079	1.2319
Nitrosomonadaceae_Ellin6067	-1.57622	0.563303
Massilia	-2.04471	0.540503
Sutterellaceae_uncultured	0.299934	-1.3461
Pseudomonas	-2.00413	0.483134
Ahniella	0.473018	-1.57691
Chthoniobacter	1.14478	1.49221
Vampirovibrionales_uncultured	0.504359	-1.82059
Verrucomicrobiaceae_uncultured	-1.9704	0.455111

FLB		
**Parameter**	**CCA1**	**CCA2**
**OTU**		
Methylobacterium-Methylorubrum	-1.42725	0.959431
Tabrizicola	0.233311	1.16744
SAR11_Clade_III	-1.16416	-0.450009
Sphingomonas	-1.00701	1.43187
Sphingorhabdus	1.16181	0.737137
Sphingomonadaceae_uncultured	-1.71898	0.926615
Polynucleobacter	0.560201	0.122269
Commamonadaceae_Other	1.12337	0.661584
Limnohabitans	0.409168	-1.03148
Candidatus_Methylopumilus	-0.810581	-0.405936
Nitrosomonadaceae_Ellin6067	0.867863	0.757994
Massilia	-1.60665	1.05585
Sutterellaceae_uncultured	-1.38875	1.11482
Arenimonas	1.40686	1.45071
Lysobacter	0.811088	1.3299

BAB: bloom attached bacteria; FLB: free living bacteria

Table S7. Permutational multivariate analysis of variance (PERMANOVA) (a) and pairwise analysis (b) to test significant differences between bacterioplankton assemblages according to period of collection.

(a) one-way PERMANOVA

Parameter	PAB (n = 21)	FLB (n = 17)
Permutation N:	999	999
Total sum of squares:	0.2611	2.352
Within-group sum of squares:	0.00933	0.8167
F:	243	13.15
p (same):	0.001	0.001

(b) Pairwise test

	PAB		FLB	
	MID	POST	MID	POST
PRE	0.003	0.003	0.009	0.021
MID		0.003		0.03

PRE: Pre-summer period; MID: Mid-summer period; POST: Post-summer period. Significant values are marked in red colour after Bonferroni correction ($p < 0.05$). PAB: particle-attached bacteria; FLB: free-living bacteria.

Table S8. Summary of significant ($p < 0.05$) positive and negative Spearman correlations (r_s) in the different bacterioplankton assemblages.

Bloom attached bacteria (BAB)

Nodes (Taxa)	Positive correlations (+) with Targets	Strength of correlation (Weight) A	B	C	D
Rhizobiales Insertae Sedis	23	2	7	10	4
Roseomonas	22	5	6	9	2
Nitrosomonadaceae Ellin6067	20	1	3	9	7
Microcystis PCC-7914	8	0	2	4	2
Vampirovibrionales	7	4	0	2	1
Sutterellaceae	6	3	0	2	1
Microscyllaceae	5	3	2	0	0
Ahniella	5	2	0	2	1
Lautropia	5	0	0	1	4
Snowella _0TU37S04	4	0	3	1	0
Aphanizomenon MD14a	3	0	2	1	0
**TOTAL**	108	20	25	41	22

Nodes (Taxa)	Negative correlations (-) with Targets	Strength of correlation (Weight) A	B	C	D
Microcystis PCC-7914	18	0	0	0	18
Microscyllaceae	7	0	0	1	6
Snowella _0TU37S04	7	0	0	1	6
Lautropia	6	0	0	4	2
Aphanizomenon MD14a	5	0	0	0	5
Vampirovibrionales	2	0	0	2	0
Ahniella	1	0	0	1	0
Nitrosomonadaceae Ellin6067	1	0	0	1	0
Rhizobiales Insertae Sedis	1	0	0	1	0
Roseomonas	1	0	0	1	0
Sutterellaceae	1	0	0	1	0
**TOTAL**	50	0	0	13	37

Free living bacteria (FLB)

Nodes (Taxa)	Positive correlations (+) with Targets	Strength of correlation (Weight) A	B	C	D
Ilumatobacteraceae__CL500-29	10	0	0	3	7
Candidatus_Limnoluna	9	0	1	1	7
Polynucleobacter	6	0	0	2	4
Vampirovibrionales	5	0	1	0	4
Deinococcus	3	0	1	0	2
SAR11_CladeIII	3	0	1	1	1
Methylobacterium-Methylorubrum	2	0	0	1	1
Sporichthyaceae_hgcI	2	0	0	1	1
Limnohabitans	2	0	0	0	2
Sphingomonas	1	0	0	1	0
**TOTAL**	43	0	4	10	29

Nodes (Taxa)	Negative correlations (-) with Targets	Strength of correlation (Weight) A	B	C	D
Ilumatobacteraceae__CL500-29	6	0	0	0	6
Sporichthyaceae_hgcI	5	0	0	0	5
SAR11_CladeIII	4	0	0	0	4
Candidatus_Limnoluna	3	0	0	0	1
Vampirovibrionales	3	0	0	0	3
Deinococcus	2	0	0	0	2
Methylobacterium-Methylorubrum	2	0	0	0	2
Limnohabitans	1	0	0	0	1
Polynucleobacter	0	0	0	0	0
Sphingomonas	0	0	0	0	0
**TOTAL**	11	0	0	0	9

Nodes represent the OTUs (Bacterial taxa, genus level) that contained the higher number of significant co-occurrences with other bacterial OTUs (represented here as the targets). The values in the table represent number of total Targets. The strength of the correlation was stratified according to the Spearman's value: (A) $r_s \geq 0.96$, (B) $0.95 \leq r_s \geq 0.90$, (C) $0.89 \leq r_s \geq 0.80$, and (D) $0.79 \leq r_s \geq 0.60$.

Table S9. Description of topological features for the construction of network analysis of PAB and FLB assemblages using the Spearman's correlation (r_s) and p significant values after Bonferroni correction.

PAB - Particle-attached bacteria

	Node topology		Edge topology			
**No.**	**Base Node**	**Target Node**	r_s	p	**S**	**W**
1	Nitrosomonadaceae_Ellin6067	Bosea	0.96	1.02E-11	+	A
2	Nitrosomonadaceae_Ellin6067	Limnohabitans	0.94	1.52E-10	+	B
3	Nitrosomonadaceae_Ellin6067	Acidibacter	0.91	7.03E-09	+	B
4	Nitrosomonadaceae_Ellin6067	Tabrizicola	0.90	4.25E-08	+	B
5	Nitrosomonadaceae_Ellin6067	Sphingorhabdus	0.89	7.51E-08	+	C
6	Nitrosomonadaceae_Ellin6067	Acetobacteraceae	0.89	8.45E-08	+	C
7	Nitrosomonadaceae_Ellin6067	Verrucomicrobiaceae	0.88	1.86E-07	+	C
8	Nitrosomonadaceae_Ellin6067	Armatimonas	0.85	1.07E-06	+	C
9	Nitrosomonadaceae_Ellin6067	Flavobacterium	0.84	2.12E-06	+	C
10	Nitrosomonadaceae_Ellin6067	Comamonadaceae	0.84	2.39E-06	+	C
11	Nitrosomonadaceae_Ellin6067	Alcaligenaceae_GKS98	0.84	2.43E-06	+	C
12	Nitrosomonadaceae_Ellin6067	Polynucleobacter	0.82	4.19E-06	+	C
13	Nitrosomonadaceae_Ellin6067	Candidatus_Methylopumilus	0.82	6.65E-06	+	C
14	Nitrosomonadaceae_Ellin6067	Aphanizomenon_NIES81	0.79	1.88E-05	+	D
15	Nitrosomonadaceae_Ellin6067	Sediminibacterium	0.79	1.92E-05	+	D
16	Nitrosomonadaceae_Ellin6067	Nitrosomonadaceae_966-1	0.79	2.35E-05	+	D
17	Nitrosomonadaceae_Ellin6067	Arenimonas	0.77	4.27E-05	+	D
18	Nitrosomonadaceae_Ellin6067	Chloroflexi_SL56	0.74	1.10E-04	+	D
19	Nitrosomonadaceae_Ellin6067	Sphingomonas	0.68	7.30E-04	+	D
20	Nitrosomonadaceae_Ellin6067	Pseudomonas	0.64	1.69E-03	+	D
21	Nitrosomonadaceae_Ellin6067	Pseudanabaena_PCC-7429	0.84	1.86E-06	-	C
22	Rhizobiales_Incertae_Sedis	Tabrizicola	0.99	3.39E-16	+	A
23	Rhizobiales_Incertae_Sedis	Acetobacteraceae	0.98	1.36E-15	+	A
24	Rhizobiales_Incertae_Sedis	Arenimonas	0.94	2.19E-10	+	B

FLB - Free-living bacteria

	Node topology		Edge topology			
**No.**	**Base Node**	**Target Node**	r_s	p	**S**	**W**
1	Candidatus_Limnoluna	Candidatus_Planktoluna	0.91	3.09E-07	+	B
2	Candidatus_Limnoluna	Arenimonas	0.89	2.36E-06	+	C
3	Candidatus_Limnoluna	Polynucleobacter	0.77	2.93E-04	+	D
4	Candidatus_Limnoluna	Sediminibacterium	0.71	1.31E-03	+	D
5	Candidatus_Limnoluna	Alcaligenaceae_GKS98	0.70	1.66E-03	+	D
6	Candidatus_Limnoluna	Sphingorhabdus	0.67	3.19E-03	+	D
7	Candidatus_Limnoluna	Nitrosomonadaceae_Ellin6067	0.60	1.08E-02	+	D
8	Candidatus_Limnoluna	Staphylococcaceae_Staphylococcus	0.58	1.48E-02	+	D
9	Candidatus_Limnoluna	Tabrizicola	0.49	4.79E-02	+	D
10	Candidatus_Limnoluna	SAR11_CladeIII	-0.56	1.97E-02	-	D
11	Candidatus_Limnoluna	Candidatus_Methylopumilus	-0.57	1.58E-02	-	D
12	Candidatus_Limnoluna	Candidatus_Planktophila	-0.70	1.61E-03	-	D
13	Ilumatobacteraceae__CL500-29	Candidatus_Limnoluna	0.84	2.32E-05	+	C
14	Ilumatobacteraceae__CL500-29	Sediminibacterium	0.84	2.60E-05	+	C
15	Ilumatobacteraceae__CL500-29	Arenimonas	0.82	5.18E-05	+	C
16	Ilumatobacteraceae__CL500-29	Sphingorhabdus	0.72	1.17E-03	+	D
17	Ilumatobacteraceae__CL500-29	Candidatus_Planktoluna	0.70	1.85E-03	+	D
18	Ilumatobacteraceae__CL500-29	Alcaligenaceae_GKS98	0.70	1.85E-03	+	D
19	Ilumatobacteraceae__CL500-29	Polynucleobacter	0.67	3.05E-03	+	D
20	Ilumatobacteraceae__CL500-29	Nitrosomonadaceae_Ellin6067	0.65	4.76E-03	+	D
21	Ilumatobacteraceae__CL500-29	Staphylococcaceae_Staphylococcus	0.62	8.26E-03	+	D
22	Ilumatobacteraceae__CL500-29	Limnohabitans	0.48	5.00E-02	+	D
23	Ilumatobacteraceae__CL500-29	Deinococcus	-0.49	4.60E-02	-	D
24	Ilumatobacteraceae__CL500-29	Vampirovibrionales	-0.53	2.93E-02	-	D

	PAB - Particle-attached bacteria							FLB - Free-living bacteria						
	Node topology		Edge topology					Node topology		Edge topology				
No.	Base Node	Target Node	r_s	*p*	S	W	No.	Base Node	Target Node	r_s	*p*	S	W	
25	Rhizobiales_Incertae_Sedis	Armatimonas	0.94	2.68E-10	+	B	25	Ilumatobacteraceae__CL500-29	Methylobacterium-Methylorubrum	-0.53	2.72E-02	-	D	
26	Rhizobiales_Incertae_Sedis	Alcaligenaceae_GKS98	0.94	2.74E-10	+	B	26	Ilumatobacteraceae__CL500-29	Candidatus_Methylopumilus	-0.55	2.26E-02	-	D	
27	Rhizobiales_Incertae_Sedis	Limnohabitans	0.94	3.00E-10	+	B	27	Ilumatobacteraceae__CL500-29	Candidatus_Planktophila	-0.56	1.98E-02	-	D	
28	Rhizobiales_Incertae_Sedis	Polynucleobacter	0.94	4.50E-10	+	B	28	Ilumatobacteraceae__CL500-29	SAR11_CladeIII	-0.59	1.28E-02	-	D	
29	Rhizobiales_Incertae_Sedis	Bosea	0.93	1.23E-09	+	B	29	Limnohabitants	Sediminibacterium	0.60	1.03E-02	+	D	
30	Rhizobiales_Incertae_Sedis	Verrucomicrobiaceae	0.92	2.14E-09	+	B	30	Limnohabitants	Staphylococcaceae_Staphylococcus	0.48	5.01E-02	+	D	
31	Rhizobiales_Incertae_Sedis	Chloroflexi_SL56	0.89	6.79E-08	+	C	31	Limnohabitants	Sutterellaceae	-0.62	7.76E-03	-	D	
32	Rhizobiales_Incertae_Sedis	Candidatus_Methylopumilus	0.88	1.21E-07	+	C	32	Polynucleobacter	Alcaligenaceae_GKS98	0.84	2.94E-05	+	C	
33	Rhizobiales_Incertae_Sedis	Sediminibacterium	0.88	2.08E-07	+	C	33	Polynucleobacter	Arenimonas	0.80	1.22E-04	+	C	
34	Rhizobiales_Incertae_Sedis	Flavobacterium	0.87	2.72E-07	+	C	34	Polynucleobacter	Sphingorhabdus	0.79	1.39E-04	+	D	
35	Rhizobiales_Incertae_Sedis	Nitrosomonadaceae_Ellin6067	0.85	1.22E-06	+	C	35	Polynucleobacter	Nitrosomonadaceae_Ellin6067	0.72	1.07E-03	+	D	
36	Rhizobiales_Incertae_Sedis	Aphanizomenon_NIES81	0.85	1.23E-06	+	C	36	Polynucleobacter	Sediminibacterium	0.61	9.20E-03	+	D	
37	Rhizobiales_Incertae_Sedis	Nitrosomonadaceae_966-1	0.84	1.64E-06	+	C	37	Polynucleobacter	Candidatus_Planktoluna	0.60	1.04E-02	+	D	
38	Rhizobiales_Incertae_Sedis	Comamonadaceae	0.82	6.18E-06	+	C	38	SAR11_CladeIII	Candidatus_Methylopumilus	0.93	3.91E-08	+	B	
39	Rhizobiales_Incertae_Sedis	Sphingorhabdus	0.81	7.46E-06	+	C	39	SAR11_CladeIII	Massilia	0.81	8.26E-05	+	C	
40	Rhizobiales_Incertae_Sedis	Acidibacter	0.80	1.22E-05	+	C	40	SAR11_CladeIII	Candidatus_Planktophila	0.70	1.60E-03	+	D	
41	Rhizobiales_Incertae_Sedis	Sphingomonas	0.79	1.67E-05	+	D	41	SAR11_CladeIII	Staphylococcaceae_Staphylococcus	-0.49	4.78E-02	-	D	
42	Rhizobiales_Incertae_Sedis	Reyranella	0.65	1.31E-03	+	D	42	SAR11_CladeIII	Sediminibacterium	-0.50	4.14E-02	-	D	
43	Rhizobiales_Incertae_Sedis	Massilia	0.61	3.33E-03	+	D	43	SAR11_CladeIII	Arenimonas	-0.53	2.71E-02	-	D	
44	Rhizobiales_Incertae_Sedis	Prochlorothrix_PCC-9006	0.59	4.49E-03	+	D	44	SAR11_CladeIII	Candidatus_Planktoluna	-0.56	1.97E-02	-	D	
45	Rhizobiales_Incertae_Sedis	Pseudanabaena_PCC-7429	0.80	1.09E-05	-	C	45	Sporichthyaceae__hgcl	Limnohabitans	0.85	1.39E-05	+	C	
46	Roseomonas	Acetobacteraceae	0.98	4.89E-15	+	A	46	Sporichthyaceae__hgcl	Staphylococcaceae_Staphylococcus	0.63	6.20E-03	+	D	
47	Roseomonas	Tabrizicola	0.98	4.43E-14	+	A	47	Sporichthyaceae__hgcl	Vampirovibrionales	-0.49	4.39E-02	-	D	
48	Roseomonas	Bosea	0.97	1.62E-12	+	A	48	Sporichthyaceae__hgcl	Methylobacterium-Methylorubrum	-0.53	2.95E-02	-	D	
49	Roseomonas	Limnohabitans	0.96	3.39E-12	+	A	49	Sporichthyaceae__hgcl	Sutterellaceae	-0.56	1.84E-02	-	D	
50	Roseomonas	Rhizobiales_Incertae_Sedis	0.96	4.04E-12	+	A	50	Sporichthyaceae__hgcl	Tabrizicola	-0.57	1.72E-02	-	D	
51	Roseomonas	Nitrosomonadaceae_Ellin6067	0.93	7.86E-10	+	B	51	Sporichthyaceae__hgcl	Hyphomonadaceae_UKL13-1	-0.57	1.65E-02	-	D	

PAB - Particle-attached bacteria

No.	Base Node	Target Node	r_s	*p*	S	W
	**Node topology**		**Edge topology**			
52	Roseomonas	Armatimonas	0.93	1.41E-09	+	B
53	Roseomonas	Verrucomicrobiaceae	0.92	2.92E-09	+	B
54	Roseomonas	Alcaligenaceae_GKS98	0.92	5.08E-09	+	B
55	Roseomonas	Polynucleobacter	0.91	6.60E-09	+	B
56	Roseomonas	Arenimonas	0.90	3.10E-08	+	B
57	Roseomonas	Acidibacter	0.88	1.11E-07	+	C
58	Roseomonas	Candidatus_Methylopumilus	0.88	1.30E-07	+	C
59	Roseomonas	Aphanizomenon_NIES81	0.88	2.08E-07	+	C
60	Roseomonas	Sphingorhabdus	0.87	2.43E-07	+	C
61	Roseomonas	Comamonadaceae	0.87	2.99E-07	+	C
62	Roseomonas	Sediminibacterium	0.86	5.52E-07	+	C
63	Roseomonas	Chloroflexi_SL56	0.86	7.31E-07	+	C
64	Roseomonas	Flavobacterium	0.85	1.35E-06	+	C
65	Roseomonas	Nitrosomonadaceae_966-1	0.81	1.05E-05	+	C
66	Roseomonas	Sphingomonas	0.74	1.25E-04	+	D
67	Roseomonas	Pseudomonas	0.59	4.52E-03	+	D
68	Roseomonas	Pseudanabaena_PCC-7429	0.87	3.40E-07	-	C
69	Snowella_0TU37S04	Chthoniobacter	0.95	5.72E-11	+	B
70	Snowella_0TU37S04	Caulobacter	0.94	1.17E-10	+	B
71	Snowella_0TU37S04	Planctomycetota_vadinHA49	0.91	1.70E-08	+	B
72	Snowella_0TU37S04	Aphanizomenon_MDT14a	0.81	6.90E-06	+	C
73	Snowella_0TU37S04	Rhizobiales_Incertae_Sedis	0.59	4.48E-03	-	D
74	Snowella_0TU37S04	Sphingomonas	0.60	4.18E-03	-	D
75	Snowella_0TU37S04	Prochlorothrix_PCC-9006	0.66	1.26E-03	-	D
76	Snowella_0TU37S04	Gemmatimonas	0.68	7.45E-04	-	D
77	Snowella_0TU37S04	Flavobacterium	0.74	1.07E-04	-	D

FLB - Free-living bacteria

No.	Base Node	Target Node	r_s	*p*	S	W
	**Node topology**		**Edge topology**			
52	Vampirovibrionales	Sutterellaceae	0.91	4.78E-07	+	B
53	Vampirovibrionales	Methylobacterium-Methylorubrum	0.67	3.25E-03	+	D
54	Vampirovibrionales	Hyphomonadaceae_UKL13-1	0.65	5.00E-03	+	D
55	Vampirovibrionales	Sphingomonas	0.58	1.42E-02	+	D
56	Vampirovibrionales	Deinococcus	0.53	2.78E-02	+	D
57	Vampirovibrionales	Polynucleobacter	-0.50	4.02E-02	-	D
58	Vampirovibrionales	Sediminibacterium	-0.59	1.18E-02	-	D
59	Vampirovibrionales	Limnohabitans	-0.62	7.69E-03	-	D

PAB - Particle-attached bacteria							FLB - Free-living bacteria						
Node topology			Edge topology				Node topology			Edge topology			
**No.**	**Base Node**	**Target Node**	r_s	***p***	**S**	**W**	**No.**	**Base Node**	**Target Node**	r_s	***p***	**S**	**W**
78	Snowella_0TU37S04	Nitrosomonadaceae_966-1	0.79	1.90E-05	-	D							
79	Snowella_0TU37S04	Reyranella	0.84	2.26E-06	-	C							
80	Aphanizomenon_MDT14a	Planctomycetota_vadinHA49	0.95	1.13E-10	+	B							
81	Aphanizomenon_MDT14a	Caulobacter	0.91	8.30E-09	+	B							
82	Aphanizomenon_MDT14a	Chthoniobacter	0.87	3.73E-07	+	C							
83	Aphanizomenon_MDT14a	Sphingomonas	0.59	4.87E-03	-	D							
84	Aphanizomenon_MDT14a	Rhizobiales_Incertae_Sedis	0.60	4.09E-03	-	D							
85	Aphanizomenon_MDT14a	Flavobacterium	0.69	5.72E-04	-	D							
86	Aphanizomenon_MDT14a	Nitrosomonadaceae_966-1	0.73	1.86E-04	-	D							
87	Aphanizomenon_MDT14a	Reyranella	0.78	2.93E-05	-	D							
88	Microcystis_PCC-7914	Ahniella	0.92	2.08E-09	+	B							
89	Microcystis_PCC-7914	Sutterellaceae	0.91	6.96E-09	+	B							
90	Microcystis_PCC-7914	Sphingobacteriales_OPS_17	0.88	1.05E-07	+	C							
91	Microcystis_PCC-7914	Pseudanabaena_PCC-7429	0.88	2.03E-07	+	C							
92	Microcystis_PCC-7914	Paludibaculum	0.85	1.10E-06	+	C							
93	Microcystis_PCC-7914	Vampirovibrionales	0.84	2.33E-06	+	C							
94	Microcystis_PCC-7914	Gemmatimonas	0.63	2.43E-03	+	D							
95	Microcystis_PCC-7914	Hyphomonadaceae_UKL13-1	0.62	2.56E-03	+	D							
96	Microcystis_PCC-7914	Limnohabitans	0.61	3.24E-03	-	D							
97	Microcystis_PCC-7914	Comamonadaceae	0.62	2.98E-03	-	D							
98	Microcystis_PCC-7914	Acetobacteraceae	0.62	2.97E-03	-	D							
99	Microcystis_PCC-7914	Bosea	0.62	2.55E-03	-	D							
100	Microcystis_PCC-7914	Arenimonas	0.63	2.31E-03	-	D							
101	Microcystis_PCC-7914	Alcaligenaceae_GKS98	0.63	2.10E-03	-	D							
102	Microcystis_PCC-7914	Chloroflexi_SL56	0.64	1.93E-03	-	D							
103	Microcystis_PCC-7914	Roseomonas	0.64	1.90E-03	-	D							
104	Microcystis_PCC-7914	Verrucomicrobiaceae	0.64	1.73E-03	-	D							

PAB - Particle-attached bacteria							FLB - Free-living bacteria						
Node topology			Edge topology				Node topology			Edge topology			
**No.**	**Base Node**	**Target Node**	r_s	***p***	**S**	**W**	**No.**	**Base Node**	**Target Node**	r_s	***p***	**S**	**W**
105	Microcystis_PCC-7914	Polynucleobacter	0.64	1.72E-03	-	D							
106	Microcystis_PCC-7914	Armatimonas	0.65	1.47E-03	-	D							
107	Microcystis_PCC-7914	Nitrosomonadaceae_Ellin6067	0.65	1.43E-03	-	D							
108	Microcystis_PCC-7914	Sediminibacterium	0.66	1.09E-03	-	D							
109	Microcystis_PCC-7914	Acidibacter	0.67	8.73E-04	-	D							
110	Microcystis_PCC-7914	Candidatus_Methylopumilus	0.69	6.04E-04	-	D							
111	Microcystis_PCC-7914	Aphanizomenon_NIES81	0.70	4.27E-04	-	D							
112	Microcystis_PCC-7914	Rhodoferax	0.74	1.29E-04	-	D							
113	Microcystis_PCC-7914	Lautropia	0.74	1.09E-04	-	D							

Base nodes represent the OTUs (Bacterial taxa, genus level) that contained the higher number of significant co-occurrences with other bacterial OTUs (represented here as the targets). r_s = Spearman's correlation. Only significant co-occurrences were added ($p < 0.05$). The correlation was categorized according to the symbol (S), as a positive (+) or negative (-) relationship, and the relative weight (W) of the Spearman's value: (A) $r_s \geq 0.96$, (B) $0.95 \leq r_s \geq 0.90$, (C) $0.89 \leq r_s \geq 0.80$, and (D) $0.79 \leq r_s \geq 0.60$.

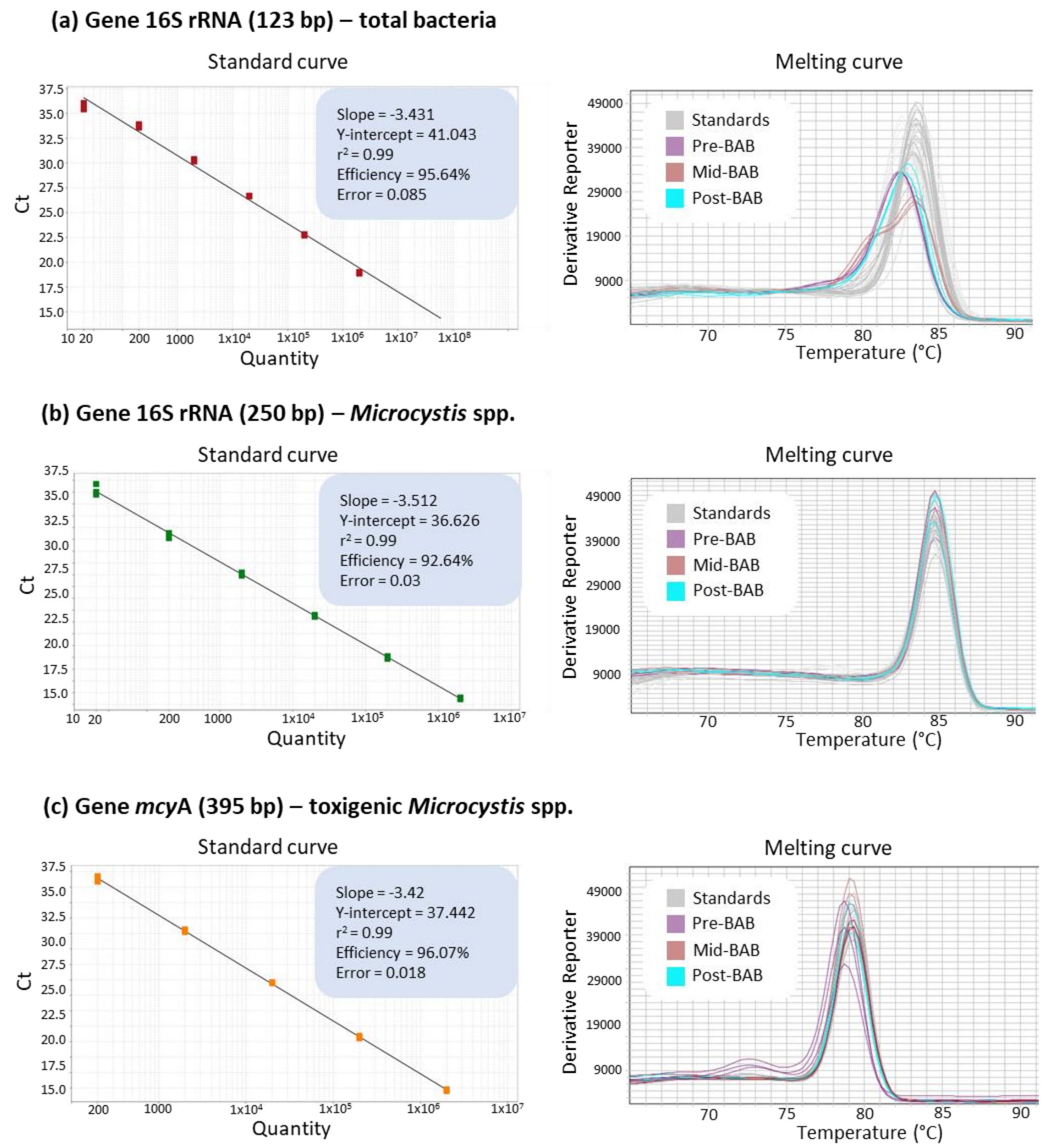

Fig S2. Standard curve and melting curve analysis of the different qPCR assays performed in the present study.
